# EGCG‐LYS Fibrils‐Mediated CircMAP2K2 Silencing Decreases the Proliferation and Metastasis Ability of Gastric Cancer Cells in Vitro and in Vivo

**DOI:** 10.1002/advs.202304075

**Published:** 2023-09-26

**Authors:** Jiaqi Dong, Zhousan Zheng, Mi Zhou, Yunfei Wang, Jiajie Chen, Junjie Cen, Tiefeng Cao, Taowei Yang, Yi Xu, Guannan Shu, Xuanxuan Lu, Yanping Liang

**Affiliations:** ^1^ Department of Oncology The First Affiliated Hospital of Sun Yat‐sen University No. 58, Zhongshan Road II Guangzhou 510080 P. R. China; ^2^ The 10th Affiliated Hospital of Southern Medical University (Dongguan People's Hospital) Southern Medical University No. 78, Wandao Road Dongguan 523059 P. R. China; ^3^ State Key Laboratory of Oncology in South China Sun Yat‐sen University Cancer Center No. 651, Dongfeng East Road Guangzhou 510060 P. R. China; ^4^ Department of Pediatrics The First Affiliated Hospital of Sun Yat‐sen University No. 58, Zhongshan Road II Guangzhou 510080 P. R. China; ^5^ Department of Urology The First Affiliated Hospital of Sun Yat‐sen University No. 58, Zhongshan Road II Guangzhou 510080 P. R. China; ^6^ Department of Gynecology The First Affiliated Hospital of Sun Yat‐sen University No. 58, Zhongshan Road II Guangzhou 510080 P. R. China; ^7^ Department of Food Science and Engineering Jinan University No. 601, West Huangpu Avenue Guangzhou 510632 P. R. China; ^8^ Department of Laboratory Medicine The First Affiliated Hospital of Sun Yat‐sen University No. 58, Zhongshan Road II Guangzhou 510080 P. R. China

**Keywords:** circRNAs, gastric cancer, nanocarriers, small interfering RNA

## Abstract

Aberrant expression of circular RNAs (circRNAs) has been reported to play an important biological regulatory role in gastric cancer (GC). For the purpose of silencing cancer‐related genes, a new approach for cancer treatment using nanocarriers to deliver siRNA has been proposed. In this study, abundantly expressed circMAP2K2 (hsa_circRNA_102415) is identified in GC cells. CircMAP2K2 regulates the PCBP1/GPX1 axis through proteasome‐mediated degradation, which further mediates the activation of the AKT/GSK3β/epithelial‐to‐mesenchymal transition (EMT) signaling pathway and enhances the proliferation and metastatic ability of GC cells. To establish novel GC treatment, epigallocatechin‐3‐gallate‐lysozyme (EGCG‐LYS) fibrils are synthesized, and in vitro experiments demonstrate that EGCG‐LYS has a higher siRNA delivery efficiency than Lipofectamine 2000 (lipo2000), which effectively silences the expression of circMAP2K2. Further studies show that EGCG‐LYS carrying siRNA can successfully achieve lysosome escape, which allows it to be located in the cytoplasm to achieve post‐transcriptional gene silencing. In addition, EGCG‐LYS carrying si‐circMAP2K2 has good circulating stability, excellent biosafety and antitumor ability in vivo. The EGCG‐LYS fibrils delivery system provides a new tool and approach for the treatment of GC.

## Graphical abstract

CircMAP2K2 regulates the PCBP1/GPX1 axis through proteasome‐mediated degradation, resulting in an epithelial‐to‐mesenchymal transition (EMT)‐like phenotype, and activated AKT/GSK3β signaling pathway enhances proliferation and metastasis of gastric cancer. In addition, EGCG‐LYS/si‐circMAP2K2 nanocarrier delivery system of small interfering RNA can effectively and safely deliver siRNA system to the tumor and has the function of knocking down target genes, providing a new tool and approach for the treatment of gastric cancer.

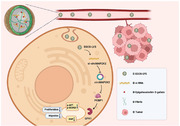

## 1 Introduction

Gastric cancer (GC) is considered to be one of the most common malignancies worldwide, with mortality that ranks among the top three cancer‐related diseases.^[^ ^1^, ^2^ ^]^ However, there are differences in the epidemiology, histopathology, biological characteristics, and treatment of gastric cancer in different countries. Gastric cancer is the second most common cancer and the leading cause of cancer death in China. The mortality to incidence ratio of gastric cancer in China was 0.845, and the 5‐year prevalence rate was 27.6/100000, both of which were higher than the rates in most developed countries.^[^ ^3^ ^]^ The incidence of early‐onset gastric cancer in young people has been increasing in recent years,^[^ ^4^ ^]^ indicating that the incidence of gastric cancer is exhibiting a trend for younger people, which also makes the challenge of gastric cancer treatment even more severe. Although research on the biology of gastric cancer is gradually deepening, surgery remains the first choice for the treatment of gastric cancer.^[^ ^5^ ^]^ Endoscopic resection is the current treatment for most cases of early gastric cancer.^[^ ^6^ ^]^ Preoperative chemotherapy may increase the chance of radical resection and eliminate early spread of the tumor.^[^ ^7^ ^]^ In patients with locally advanced unresectable or metastatic gastric cancer, the use of chemotherapy can improve their survival and quality of life.^[^ ^8^ ^]^ The application of high‐throughput gene sequencing and other technologies have facilitated intensive exploration of genomic and epigenomic changes related to gastric cancer. Genetic and epigenetic factors related to the pathogenesis of gastric cancer include gene mutations, chromosome aberrations, differential gene expression, and epigenetic changes.^[^ ^9^ ^]^ In addition, gene sequencing and correlation analyses have discovered more key signaling pathways regulating gastric cancer. These studies have expanded our understanding of gastric cancer at the molecular level and have potential guiding significance for future targeted therapy of gastric cancer.

Non‐coding RNAs (ncRNAs) lack the potential to encode proteins, but are instrumental in gene network regulation and oncogenesis and development.^[^ ^10^ ^]^ Thus, ncRNAs can function as oncodrivers or tumor suppressors to regulate cellular biological responses in cancers. Consequently, targeting ncRNAs is a very promising therapeutic direction to fight against cancer. The most studied types of “classical ncRNAs” are microRNAs (miRNAs),^[^ ^11^ ^]^ long non‐coding RNAs (lncRNAs),^[^ ^12^ ^]^ and circular RNAs (circRNAs).^[^ ^13^ ^]^ Among them, circRNA is a covalently closed ring where the 3′ end and the 5′ end are joined together, and this structure results in the high stability of circRNA.^[^ ^14^ ^]^

The RNA interference (RNAi) pathway is responsible for regulating mRNA stability and translation in human cells.^[^ ^15^ ^]^ Small double‐stranded RNA molecules can effectively achieve RNAi silencing of specific genes.^[^ ^16^ ^]^ Continuous development of technology and scientific research has markedly expanded the practical application of RNA‐based therapy in clinical practice, among which an important method is the use of antisense oligonucleotides and small interfering RNA (siRNA) for treatment.^[^ ^17^ ^]^ SiRNAs can reduce the level of target genes expression in cancer cells.^[^ ^18^ ^]^ Crucially, we need to deliver the siRNA molecule into the target cell to activate the RNAi pathway. However, siRNA molecules are large and hydrophilic and thus cannot diffuse across cell membranes. To this end, it is necessary to help cells take up siRNA by chemically modification or delivering materials.^[^ ^19^ ^]^ Furthermore, when delivering drugs systematically, it is necessary to design delivery systems that have addressed the various physiological obstacles to the arrival of siRNA at its site of action, including stability of antiserum nuclease, exit from blood vessels into target tissues, entry into cells, and prevention of renal clearance.^[^ ^20^ ^]^ The use of nanocarriers to deliver siRNAs that can silence cancer‐related genes is a new approach for cancer therapy.

During our preliminary screening for siRNA nanocarriers, it was discovered that lysozyme (LYS) could be a promising candidate. LYS is a monomeric protein from egg white with a molecular weight of 14.3kDa.^[^ ^21^ ^]^ Under certain conditions, LYS forms rod‐like protein fibrils, which are supramolecular aggregates of proteins or peptides with high length‐to‐diameter ratio, similar to the classic amyloid protein fibrils.^[^ ^22^ ^]^ Despite their small thickness (generally <20 nm), these protein fibrils possess strong mechanical strength comparable to that of steel and silk.^[^ ^23^ ^]^ It has been reported that these protein fibrils can retain their stable structure under harsh conditions like low pH and long‐term heat treatment,^[^ ^22^ ^]^ and most protein fibrils are biocompatible and non‐cytotoxic.^[^ ^24^ ^]^ Also, the LYS fibrils exhibit positive charge, which is optimal for siRNA‐binding and tumor targeting through electrostatic interactions, given that siRNA and many types of cancer cells^[^ ^25^ ^]^ are negatively charged. Moreover, LYS fibrils can readily cross cell membrane via various mechanisms.^[^ ^26^ ^]^ In addition, LYS fibrils have well‐documented anti‐cancer, antibacterial characteristics.^[^ ^27^, ^28^ ^]^ In neuroblastoma, the lysozyme fibrils achieve anti‐cancer effect by inducing cell death via different mechanisms involving apoptotic and necrotic pathways.^[^ ^28^ ^]^ Because of these superior characteristics of LYS fibrils, it was chosen as the main nanocarrier candidate for siRNA delivery in this study. Moreover, according to our previous research, epigallocatechin‐3‐gallate (EGCG), a major bioactive compound derived from green tea, has considerable anti‐tumor effect.^[^ ^29^ ^]^ Thus, an EGCG‐LYS nanoparticle complex was synthesized and used in this study.

In this study, two published circRNAs microarray datasets of gastric cancer from the Gene Expression Omnibus data repository (GEO, https://www.ncbi.nlm.nih.gov/gds/) were analyzed using a bioinformatics approach. The screening revealed that circMAP2K2 may play a role in promoting the occurrence and development of gastric cancer. Experiments conducted in vitro and in vivo demonstrated that circMAP2K2 regulates the PCBP1/GPX1 axis through proteasome‐mediated degradation, resulting in activation of the AKT/GSK3β signaling pathway and epithelial‐to‐mesenchymal transition (EMT)‐like phenotype, thereby enhancing the proliferation, migration, and invasion of gastric cancer cells. Next, the therapeutic effect of epigallocatechin‐3‐gallate‐lysozyme (EGCG‐LYS) fibrils carrying si‐circMAP2K2 in tumors was explored as a potential strategy to reduce the expression of circMAP2K2 gene‐targeting tumors and thus inhibit tumor proliferation and metastasis. EGCG‐LYS fibrils were developed through a one‐step heating process under certain pH conditions, and negatively charged siRNAs were absorbed into the positively charged amnio groups of the EGCG‐LYS fibrils via a simple mixing procedure. It was hypothesized that beneficial properties of EGCG and LYS, such as anticancer functions, would enhance the cancer therapy effects of siRNA. Findings from the study may provide clues for the design of effective development programs and could increase the likelihood of clinical success.

## 2 Results

### 2.1 Oncogenic circMAP2K2 was Highly Expressed in GC Cell Lines

**Figure 1:**
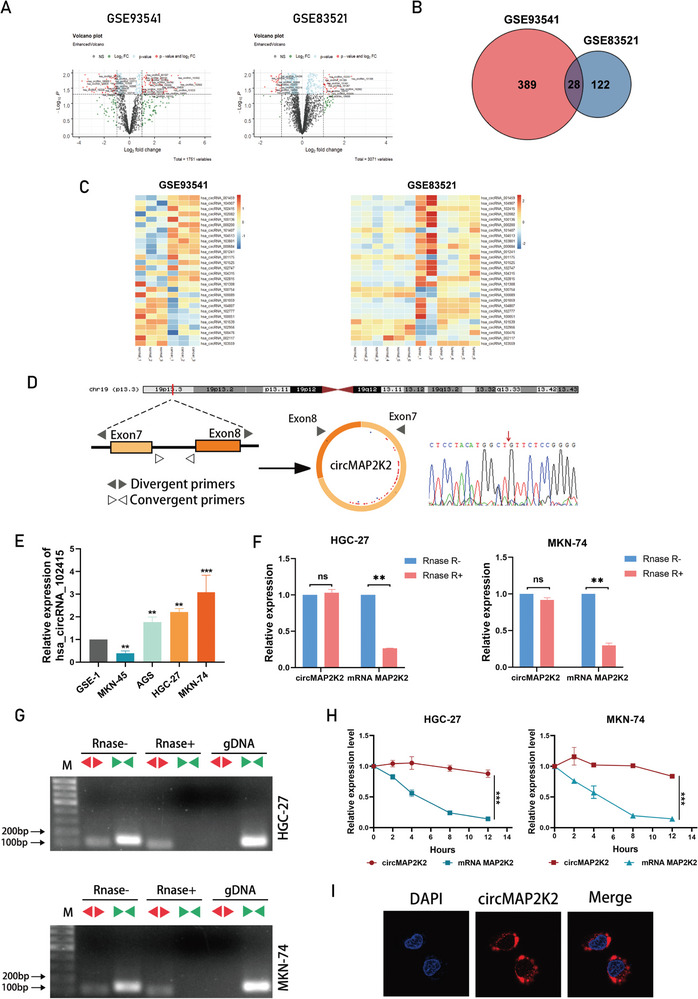
Oncogenic circMAP2K2 was highly expressed in GC cell lines. A) Volcano plot of GSE93541 and GSE83521. Red dots indicate significantly upregulated or downregulated circRNAs. B) Venn diagram of the two data sets. The overlapping area indicates significant circRNAs in both datasets. C) Heatmaps of the two datasets. The red color represents high expression while the blue color represents low expression. D) The back‐splicing of circMAP2K2 in circMAP2K2‐PCR products was confirmed by Sanger sequencing. The red arrow indicates the specific connection point. E) qRT‐PCR was used to detect the abundance of circMAP2K2 in normal gastric epithelial cell line GSE‐1 and gastric cancer cell lines MKN‐45, AGS, MKN‐74, and HGC‐27. GAPDH as internal control. Expression was normalized to GSE‐1. F) After RNase treatment, the stability of circMAP2K2 and linear MAP2K2 was determined by qRT‐PCR. G) Electrophoresis experiments examined the expression levels of the back‐splicing and canonical forms of MAP2K2 in cDNA (with or without RNase R treatment) and gDNA from GC cells. H) qRT‐PCR measuring the stability of circMAP2K2 and linear MAP2K2 in HGC‐27 and MKN‐74 cells treated with actinomycin D. I) The cellular localization of circMAP2K2 detected by FISH was mostly in the cytoplasm. The nuclei were labeled with DAPI dye. All data are presented as the mean ± SEM. n = 3. *p < 0.05, **p < 0.01, ***p < 0.001.

With the purpose of investigating the role of circRNAs in the development of gastric cancer, two GEO datasets (GSE93541 and GSE83521) of circRNA microarrays from human tissue samples were analyzed. After identifying differentially expressed genes, circRNAs with a log2 fold‐change >1 or <−1, and *P* <0.05 were selected (**Figure** **1**A). Common altered circRNAs in the two data sets that represent a consistent pattern of regulation in GC were then extracted, yielding a subset of 28 circRNAs (Figure 1B). At this time, all of these 28 significantly altered circRNAs were selected (Table S2, Supporting Information) and a heat map of the expression of these circRNAs in both datasets was generated (Figure 1C). Among them, the expression differences of hsa_circRNA_001459, hsa_circRNA_102415, hsa_circRNA_102082, and hsa_circRNA_000200 were the most significant in both data sets. Next, the expression patterns of the above four circRNAs were assessed by qRT‐PCR, and only hsa_circRNA_102415 and hsa_circRNA_000200 were upregulated in gastric cancer cell lines compared with normal gastric mucosal epithelial cells (Figure 1E; Figure S1, Supporting Information). The circRNA hsa_circRNA_000200 was already previously reported to promote the progression of gastric cancer.^[^ ^30^ ^]^ Therefore, hsa_circRNA_102415 was selected as the research target for this study.

CircMAP2K2 (Arraystar ID: hsa_circRNA_102415, circBase ID: hsa_circ_0007376), which was identified as the circRNA of interest, is derived from the MAP2K2 gene on chr19:4101016‐4101278, resulting from back‐splicing of exons 7 and 8 (177 bp). Sanger sequencing was conducted to confirm the back‐splicing junctions of circMAP2K2 (Figure 1D). We have confirmed that the expression levels of circMAP2K2 in the MKN‐45, AGS, MKN‐74 and HGC‐27 GC cell lines relative to gastric epithelial cells GSE‐1 cells (Figure 1E). MKN‐74 cells showed the highest expression of circMAP2K2, while HGC‐27 cells ranked second. However, the expression of circMAP2K2 was lower in MKN‐45 cells compared with that of normal gastric epithelial cells. Thus, MKN‐74 cells, HGC‐27 cells, and MKN‐45 gastric cancer cells were selected to investigate the underlying biological traits of circMAP2K2.

First, qRT‐PCR was performed using two sets of primers, with the partial primer amplifying only the linear form and the divergent primer amplifying only the circular form of MAP2K2. CircMAP2K2, but not linear MAP2K2, was resistant to RNase R digestion as determined by qRT‐PCR, indicating that the back‐splicing product was not derived from gene trans‐splicing or genome rearrangement (Figure 1F). PCR and agarose gel electrophoresis confirmed that circMAP2K2 could only be amplified from cDNA and not from gDNA (Figure 1G). Also, circMAP2K2 had a longer half‐life than MAP2K2 mRNA and its expression was not downregulated by actinomycin D treatment (Figure 1H). Based on the results of FISH analysis of circMAP2K2's subcellular localization in MKN‐74 cells, we can clearly see that the primary localization of circMAP2K2 is in the cytoplasm. (Figure 1I).

### 2.2 CircMAP2K2 Promotes GC Cells Proliferation and Metastasis

**Figure 2:**
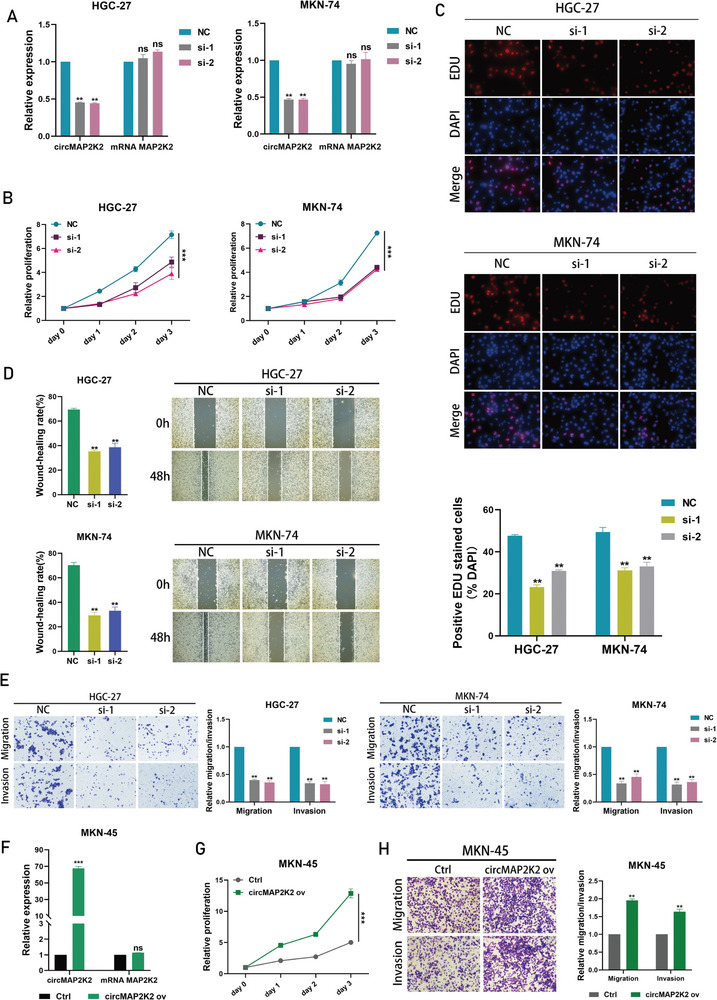
CircMAP2K2 promotes GC cells proliferation and metastasis. A) The knockdown efficiency of HGC‐27 and MKN‐74 cells was determined by qRT‐PCR. GAPDH as internal control. Expression was normalized to NC group. B) CCK8 assay assessing the proliferation of HGC‐27 and MKN‐74 cells transfected with circMAP2K2 siRNAs or siNC. Proliferation rate was normalized to day 0. C) EdU assay assessing the proliferation of HGC‐27 and MKN‐74 cells transfected with circMAP2K2 siRNAs or siNC. D) Wound healing assay assessing the migration potential of HGC‐27 and MKN‐74 cells transfected with circMAP2K2 siRNAs or siNC. E) Transwell assay assessing the migration and invasion potential of HGC‐27 and MKN‐74 cells transfected with circMAP2K2 siRNAs or siNC. F) qRT‐PCR was used to detect the expression level of circMAP2K2 in MKN‐45 cell lines transfected with circMAP2K2 overexpression plasmid or control vector. GAPDH as internal control. Expression was normalized to control (Ctrl) group. G) CCK8 assay assessing the proliferation of MKN‐45 cells transfected with circMAP2K2 overexpression plasmid or control (Ctrl) vector. Proliferation rate was normalized to day 0. H) Transwell assay assessing the migration and invasion of MKN‐45 cells transfected with circMAP2K2 overexpression plasmid or control (Ctrl) vector.

To evaluate the biological role of circMAP2K2 in gastric cancer, some routine functional analyses were performed. To manipulate the expression of circMAP2K2, siRNAs targeting the back‐splicing junction were constructed. Predictably, the engineered siRNA significantly silenced circMAP2K2 expression in the two GC cell lines (HGC‐27 and MKN‐74) with the highest circMAP2K2 expression, while the expression of the linear form of MAP2K2 mRNA was not altered (**Figure** **2**A). Subsequently, CCK‐8 and EdU assays were performed to determine the proliferation ability of gastric cancer cells, and a transwell assay and wound healing assay were employed to determine the metastatic ability of gastric cancer cells. The CCK‐8 assay showed that circMAP2K2 silencing significantly inhibited the cell proliferation rate (Figure 2B), and this finding was further confirmed by the EdU assay (Figure 2C). Knockdown of circMAP2K2 also successfully reduced the migration and invasion capacities of HGC‐27 and MKN‐74 cells (Figure 2D,E). In addition, we designed the construction of circMAP2K2 overexpression vector, which resulted in a significant up‐regulation of circMAP2K2 expression in the MKN‐45, a cell line with a relatively low abundance of circMAP2K2 among the three GC cell lines used in this study (Figure 2F). Consistent with the role of knockdown experiments in tumorigenesis, overexpression of circMAP2K2 via the lentiviral vector significantly increased cell proliferation (CCK‐8 and EdU), migration, invasion, and wound healing in the MKN‐45 cell line (Figure 2G,H; Figure S2A,  S2B, Supporting Information).

### 2.3 EIF4A3 Induced circMAP2K2 Expression in GC Cells

During exploration of the mechanism of circMAP2K2 upregulation in gastric cancer, Circular RNA Interactome (https://circinteractome.nia.nih.gov/) analysis revealed that EIF4A3 had seven binding sites in the upstream region of the MAP2K2 mRNA transcript, which were concentrated in a region of 800 bases. The predicted binding sites were divided into four blocks on average: a, b, c, and d (Figure S3A, Supporting Information). In the pull‐down assay of MAP2K2 mRNA, it was confirmed that EIF4A3 protein was more significantly enriched by the pull‐down of MAP2K2 mRNA but not in the control (Figure S3B, Supporting Information). For the sake of detecting whether EIF4A3 bind to certain predicted regions on the MAP2K mRNA transcript, we performed RIP assay using EIF4A3 antibodies and then qRT‐PCR assay using primers designed for the four binding regions (a, b, c, and d). Fragments a, b, c, and d were all enriched in EIF4A3 precipitates (Figure S3B, Supporting Information). Next, five RNA transcripts that contained different MAP2K2 sequences were constructed, and the RNA pull‐down assay was repeated. The results showed that the upstream sequence of circMAP2K2 contained binding sites to EIF4A3, further confirming the interaction between EIF4A3 and MAP2K2 mRNA (Figure S3C, Supporting Information). Subsequently, knockdown of EIF4A3 expression was found to reduce circMAP2K2 expression in MKN‐74 and HGC‐27 cells, while overexpression of EIF4A3 will lead to increased expression of circMAP2K2 (Figure S3D,  S3E, Supporting Information).

### 2.4 CircMAP2K2 Regulates PCBP1 Function Through Proteasome‐Mediated Degradation

**Figure 3:**
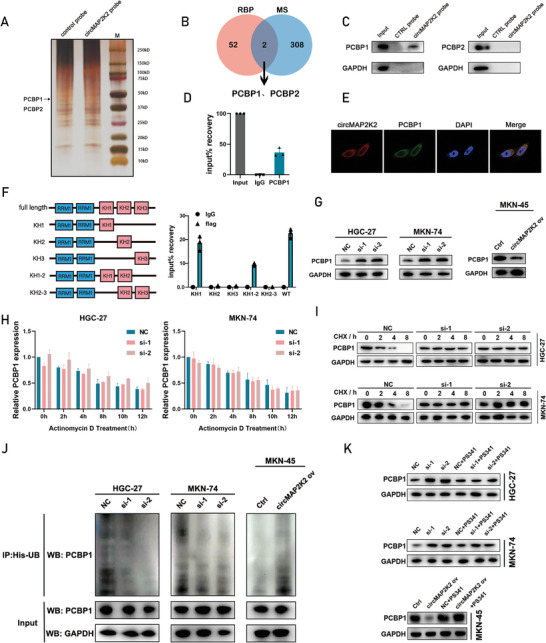
CircMAP2K2 regulates PCBP1 function through proteasome mediated degradation. A) Protein extracts from MKN‐74 cells were used to identify circMAP2K2 protein complexes pulled down by circMAP2K2‐ligation probes. The protein was visualized with silver staining. B) Overlap of proteins obtained from mass spectrometry and RBPmap database analysis. C) Western blot after RNA pull‐down analysis revealed that PCBP1, but not PCBP2, was pulled down by circMAP2K2 probe. GAPDH was used as internal control. D) qRT‐PCR after RIP assay (PCBP1 antibody) showed that circMAP2K2 was recruited from PCBP1 protein in MKN‐74 cell lysates. IgG antibody was used as a control. E) The co‐localization of circMAP2K2 and PCBP1 protein in the cytoplasm was detected by IF‐FISH. Nuclei were labeled with DAPI dye. F) Left: schematic shows the structure of the RNA binding domain in PCBP1 protein and the structure of PCBP1 truncation. Right: the relative enrichment identified by RIP assay represents PCBP1 truncated correlated circMAP2K2 levels. G) Western blot shows the protein level of PCBP1 after circMAP2K2 knockdown and overexpression. GAPDH was used as internal control. H) Actinomycin D treatment and qRT‐PCR were used to detect the effect of circMAP2K2 knockdown on the mRNA stability of PCBP1 in HGC‐27 and MKN‐74 cells. I) Western blot analysis of PCBP1 in HGC‐27 and MKN‐74 cells treated with transcription inhibitor CHX (200 µg mL^−1^). The protein stability of PCBP1 was measured. GAPDH was used as internal control. J) Immunoprecipitation (IP) analysis of ubiquitinated PCBP1 in HGC‐27, MKN‐74, and MKN‐45 cells. Cell lysates with knockdown or overexpression of circMAP2K2 were used to pull down his‐UB coupling proteins using his‐beads. The ubiquitination level of PCBP1 was determined by anti‐PCBP1 antibody. K) Proteasome inhibitor PS341 blocked the effect of circMAP2K2 knockdown on the stability of PCBP1 protein. GAPDH was used as internal control.

RNA pull‐down assays followed by mass spectrometry analysis were performed to identify circMAP2K2‐associated proteins. A total of 310 proteins interacting with circMAP2K2 were identified using this approach. In addition, the RBPmap database (http://rbpmap.technion.ac.il/index_DEV.html) was utilized to predict candidate RNA‐binding proteins (RBPs) that interact with circMAP2K2, and 54 proteins were identified (**Figure** **3**A,B). Among the identified RBPs, PCBP1 and PCBP2 were included in both the mass spectrometry analysis and the RBPmap database results (Figure 3B). As shown in the western blot after RNA pull‐down analysis, PCBP1 had higher coverage than PCBP2 (Figure 3C; Figure S4A, Supporting Information). The interaction between circMAP2K2 and PCBP1 was further confirmed by RIP assay, which showed that circMAP2K2 could bind to PCBP1 (Figure 3D). Subsequently, the colocalization of endogenously expressed circMAP2K2 and PCBP1 in the cytoplasm was determined by immunofluorescence and FISH (IF‐FISH, Figure 3E). To predict which domain of PCBP1 was involved in the interaction with circMAP2K2, the catRAPID tool was utilized, and based on the results (Figure S4B, Supporting Information), PCBP1 mutants with individual RNA‐binding K homologous (KH) domain truncations were constructed. Consistent with the predictions, further RIP analysis revealed that only full length (FL) and KH1 were pulled down by circMAP2K2 (Figure 3F). These lines of evidence suggest that circMAP2K2 interacts with PCBP1 in GC. Furthermore, these results collectively suggested that circMAP2K2/PCBP1 formed an interacting RNA‐protein complex in the cytoplasm.

Next, the regulatory relationship between circMAP2K2 and PCBP1 at RNA and protein levels was investigated. CircMAP2K2 was found to regulate the protein level, but not the mRNA level or stability, of PCBP1 (Figure 3G; Figure S4C, Supporting Information). This suggested that circMAP2K2 may regulate PCBP1 at the post‐transcriptional level. Blocking PCBP1 protein synthesis revealed that the half‐life of PCBP1 protein, but not RNA, was markedly different between the negative control (NC) and the two groups of si‐circMAP2K2, indicating that circMAP2K2 reduced the stability of PCBP1 protein (Figure 3H,I). In addition, silencing circMAP2K2 simultaneously decreased the polyubiquitination of PCBP1 in GC cells (Figure 3J). Coincidentally, after treatment with the proteasome inhibitor PS341, the protein expression level of PCBP1 was unchanged in circMAP2K2 knockdown and circMAP2K2 overexpression cells (Figure 3K). To sum up, these results indicated that circMAP2K2 affected the proteasome‐mediated degradation and expression of PCBP1 after translation.

### 2.5 PCBP1 Inhibits the Proliferation, Invasion, and Metastasis of Gastric Cancer Cells

**Figure 4:**
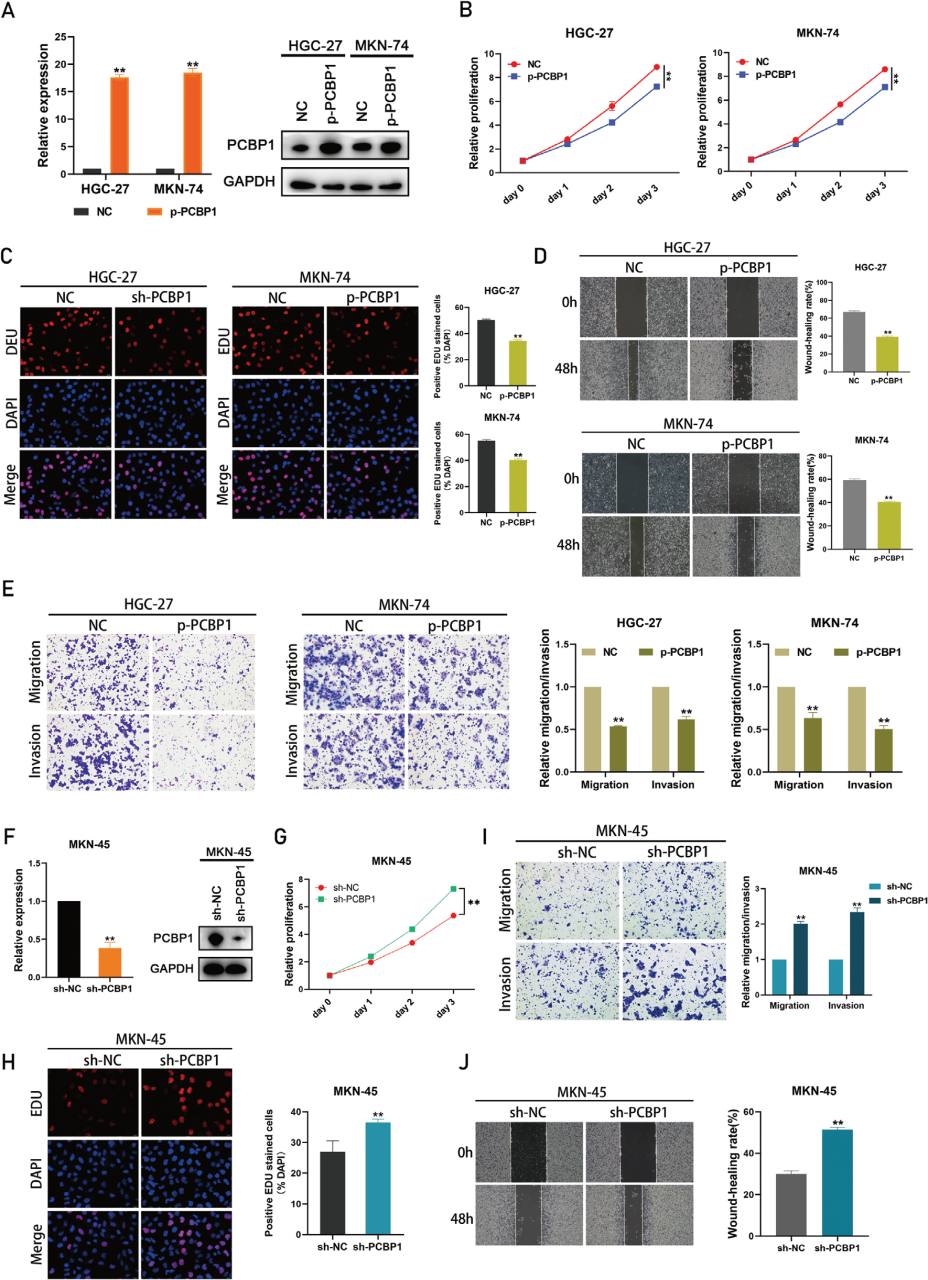
PCBP1 inhibits the proliferation, invasion and metastasis of gastric cancer cells. A) qRT‐PCR and Western blot conformed that PCBP1 was overexpressed in HGC‐27 and MKN‐74 cells. B) CCK8 assay assessing the proliferation of HGC‐27 and MKN‐74 cells transfected with PCBP1 overexpression plasmid or control vector. Proliferation rate was normalized to day 0. C) EdU assay assessing the proliferation of HGC‐27 and MKN‐74 cells transfected with PCBP1 overexpression plasmid or control vector. D) Wound healing assay assessing the migration potential of HGC‐27 and MKN‐74 cells transfected with PCBP1 overexpression plasmid or control vector. E) Transwell assay assessing the migration and invasion potential of HGC‐27 and MKN‐74 cells transfected with PCBP1 overexpression plasmid or control vector. F) qRT‐PCR and Western blot indicated that the PCBP1 gene was silenced by transfection of shRNA in MKN‐45 cells. G) CCK8 assay assessing the proliferation of MKN‐45 cells transfected with PCBP1 shRNA or shNC. Proliferation rate was normalized to day 0. H) EdU assay assessing the proliferation of MKN‐45 cells transfected with PCBP1 shRNA or shNC. I) Transwell assay assessing the migration and invasion potential of MKN‐45 cells transfected with PCBP1 shRNA or shNC. J) Wound healing assay assessing the migration potential of MKN‐45 cells transfected with PCBP1 shRNA or shNC.

It was previously reported that PCBP1 was the most downregulated protein in the metastatic tissue specimens of gastric cancer.^[^ ^31^ ^]^ Therefore, we hypothesized that PCBP1 might act as a tumor suppressor gene in gastric cancer. Consistent with this hypothesis, overexpression of PCBP1 (p‐PCBP1) in HGC‐27, and MKN‐74 cells resulted in decreased proliferation, invasion, and metastasis (**Figure** **4**A–E). Loss‐of‐function experiments were also performed, and knockdown in MKN‐45 cells showed the opposite results (Figure 4F–J). This collection of results demonstrated that PCBP1 could inhibit the malignant behavior of gastric cancer cells.

### 2.6 CircMAP2K2 Mainly Promotes the Growth and Metastasis of Gastric Cancer by Inhibiting PCBP1

**Figure 5:**
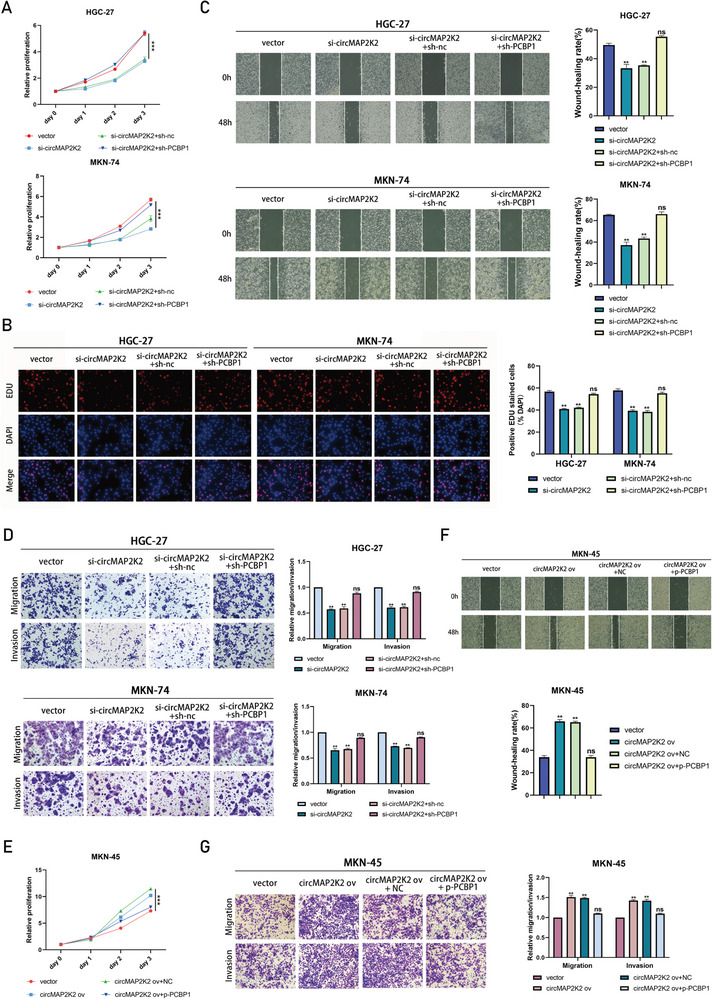
CircMAP2K2 mainly promotes the growth and metastasis of gastric cancer by inhibiting the PCBP1. A) CCK8 assay assessing the proliferation of HGC‐27 and MKN‐74 cells rescue experiment (circMAP2K2 knockdown ± PCBP1 knockdown). Proliferation rate was normalized to day 0. B) EdU assay assessing the proliferation of HGC‐27 and MKN‐74 cells rescue experiment (circMAP2K2 knockdown ± PCBP1 knockdown). C) Wound healing assay assessing the migration potential of HGC‐27 and MKN‐74 cells rescue experiment (circMAP2K2 knockdown ± PCBP1 knockdown). D) Transwell assay assessing the migration and invasion potential of HGC‐27 and MKN‐74 cells rescue experiment (circMAP2K2 knockdown ± PCBP1 knockdown). E) CCK8 assay assessing the proliferation of MKN‐45 rescue experiment (circMAP2K2 overexpression ± PCBP1 overexpression). Proliferation rate was normalized to day 0. F) Wound healing assay assessing the migration potential of HGC‐27 and MKN‐74 cells rescue experiment (circMAP2K2 overexpression ± PCBP1 overexpression). G) Transwell assay assessing the migration and invasion potential of HGC‐27 and MKN‐74 cells rescue experiment (circMAP2K2 overexpression ± PCBP1 overexpression). Labels: si‐circMAP2K2: circMAP2K2 knockdown. sh‐PCBP1: PCBP1 knockdown. circMAP2K2 ov: circMAP2K2 overexpression. p‐PCBP1: PCBP1 overexpression.

To investigate whether circMAP2K2 plays a carcinogenic role by affecting the expression of PCBP1, rescue experiments were conducted with sh‐PCBP1 and p‐PCBP1. The knockdown of circMAP2K2 expression in HGC‐27 cells and MKN‐74 cells showed that the proliferation and invasion of the two cells were effectively inhibited. However, application of sh‐PCBP1 could rescue the inhibitory effect on the cells (**Figure** **5**A,B). Consistent with the observed functional changes, results from the wound healing assay, transwell migration, and Matrigel invasion assay also showed that circMAP2K2 siRNA transfection could be rescued by knocking down PCBP1 expression in HGC‐27 and MKN‐74 cells (Figure 5C,D). Furthermore, similar to the previous experiments, p‐PCBP1 largely abolished the increased proliferation and invasion of MKN‐45 cells induced by circMAP2K2 overexpression (Figure 5E–G; Figure S5, Supporting Information). Therefore, circMAP2K2 promoted the malignant phenotype of GC mainly by activating the PCBP1 pathway.

### 2.7 Elucidation of the Potential Oncogenic Mechanism of circMAP2K2 and the Effect of Downregulation of circMAP2K2 on the Progression of GC Tumors In Vivo

**Figure 6:**
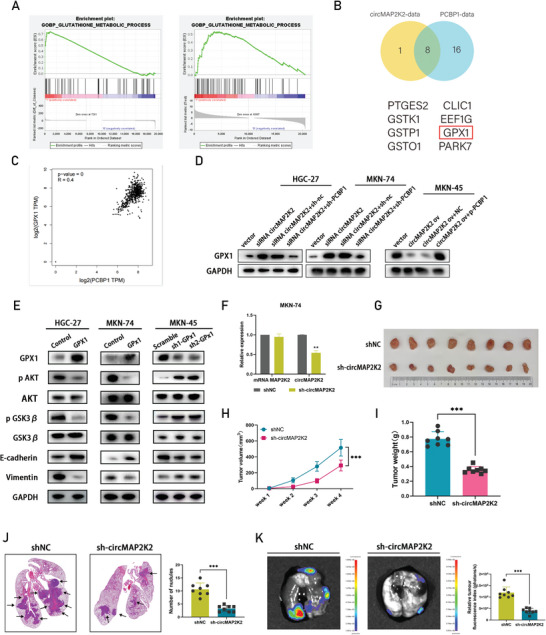
Elucidation of the potential oncogenic mechanism of circMAP2K2 and the effect of downregulation of circMAP2K2 on the progression of GC tumors in vivo. A) The gene set enrichment analysis of glutathione metabolic pathway in circMAP2K2 RNA high‐expression sequencing data and PCBP1 low‐expression TCGA patients. B) Venn diagram shows the key gene set of the co‐enrichment pathway of circMAP2K2 RNA high expression and PCBP1 low expression. The overlapping portion indicates mutual genes in two datasets. C) Correlation analysis between PCBP1 and GPX1 expression in gastric cancer on GEPIA website. D) Western blot analysis showed that silencing PCBP1 could eliminate the up‐regulation of GPX1 mediated by silencing circMAP2K2, and overexpression of PCBP1 could save the down‐regulation of GPX1 caused by overexpression of circMAP2K2 (Labels: si‐circMAP2K2: circMAP2K2 knockdown. sh‐PCBP1: PCBP1 knockdown. circMAP2K2 ov: circMAP2K2 overexpression. p‐PCBP1: PCBP1 overexpression). E) After knockdown and overexpression of GPX1, the expression levels of EMT markers (E‐cadherin and vimentin) and p‐AKT, AKT, p‐GSK3β, and GSK3β were detected by western blotting. GAPDH was used as the internal control. F) Establishment of a stable circMAP2K2 knockdown MKN‐74 cell line. The relative expression levels of linear MAP2K2 and circMAP2K2 were detected by qRT‐PCR. G) The representative tumor picture of the subcutaneous xenograft models. Knockdown of circMAP2K2 significantly inhibited tumor growth in vivo. H) Weekly tumor volume changes were recorded and displayed. I) Final tumor weights at week 4. J) (left) The representative HE staining picture of lung infiltration in shNC and sh‐circMAP2K2 animal groups. Arrows indicate tumor nodules. (right) The metastatic nodules of mice in both groups were observed and counted under microscope. K) (left) The representative picture of in vivo bioluminescence lung metastasis image in shNC and sh‐circMAP2K2 animal groups. (right) The quantification of bioluminescence intensity in shNC and sh‐circMAP2K2 animal groups.

According to Gene Ontology (GO) pathway enrichments (https://www.gsea‐msigdb.org/gsea/index), the circMAP2K2 RNA‐seq expressing and PCBP1 were mainly enriched in the GOBP_GLUTATHIONE_METABOLIC_PROCESS signaling pathway (**Figure** **6**A). Among the candidate genes (PTGES2, CLIC1, PARK7, GSTO1, EEF1G, GSTP1, GPX1, and GSTK1; Figure 6B), GPX1 is the only one that has been reported to be related to GC progression.^[^ ^32^ ^]^ To predict the relationship between GPX1 and PCBP1, the Gene Expression Profiling Interactive Analysis (GEPIA) database (http://gepia.cancer‐pku.cn/index.html) was utilized, and the results showed a positive correlation between PCBP1 and GPX1 levels (Figure 6C). This relationship was verified by qRT‐PCR as well as Western blot analyses following PCBP1 knockdown in HGC‐27 and MKN‐74 cells (Figure S6A,  S6B, Supporting Information). We hypothesized that circMAP2K2 might affect GPX1 by regulating PCBP1 expression. Western blotting showed that silencing PCBP1 could eliminate the upregulation of GPX1 mediated by silencing circMAP2K2, and overexpression of PCBP1 could rescue the downregulation of GPX1 caused by overexpression of circMAP2K2, which confirmed our conjecture (Figure 6D). It was previously reported that loss of GPX1 drives EMT and chemotherapy resistance by activating the AKT/GSK3β axis. Therefore, we hypothesized that the loss of GPX1 in GC cells might result in an EMT‐like phenotype and that activated AKT/GSK3β signaling is involved in this process. As shown in Figure 6E, GPX1 silencing increased the expression of phosphorylated AKT (p‐AKT), phosphorylated GSK3β (p‐GSK3β) and EMT‐related proteins. In contrast, the expression levels of these proteins were significantly decreased in GPX1 overexpression cells.

To evaluate the potential oncogenic mechanism of circMAP2K2 in vivo, a subcutaneous implantation model was established in male BALB/c nude mice. MKN‐74 cells were transfected with circMAP2K2 shRNA stably expressing luciferase, and negative control cells transfected with vector were set up at the same time (Figure 6F). Knockdown of circMAP2K2 effectively inhibited the growth rate of xenografts in vivo compared with that of the control group (Figure 6G). This was also reflected in the fact that circMAP2K2‐silenced nude mice exhibited smaller changes in tumor size and a smaller tumor weight in comparison with the control mice (Figure 6H,I). To test the effect of circMAP2K2 on gastric cancer metastasis further, a tumor metastasis model was constructed by injecting circMAP2K2‐knockdown MKN‐74 cells into mice via the tail vein. Lung metastatic sites were observed in the sh‐circMAP2K2 group compared with the shNC group. In vivo imaging of anatomical lung organs and histological examination confirmed that mice injected with circMAP2K2‐knockdown cancer cells had significantly fewer metastatic lesions in the lungs compared with the controls (Figure 6J,K).

### 2.8 EGCG‐LYS Fibrils‐Mediated circMAP2K2 Silencing Synergistically Inhibit Gastric Cancer Cells Growth In Vitro

**Figure 7:**
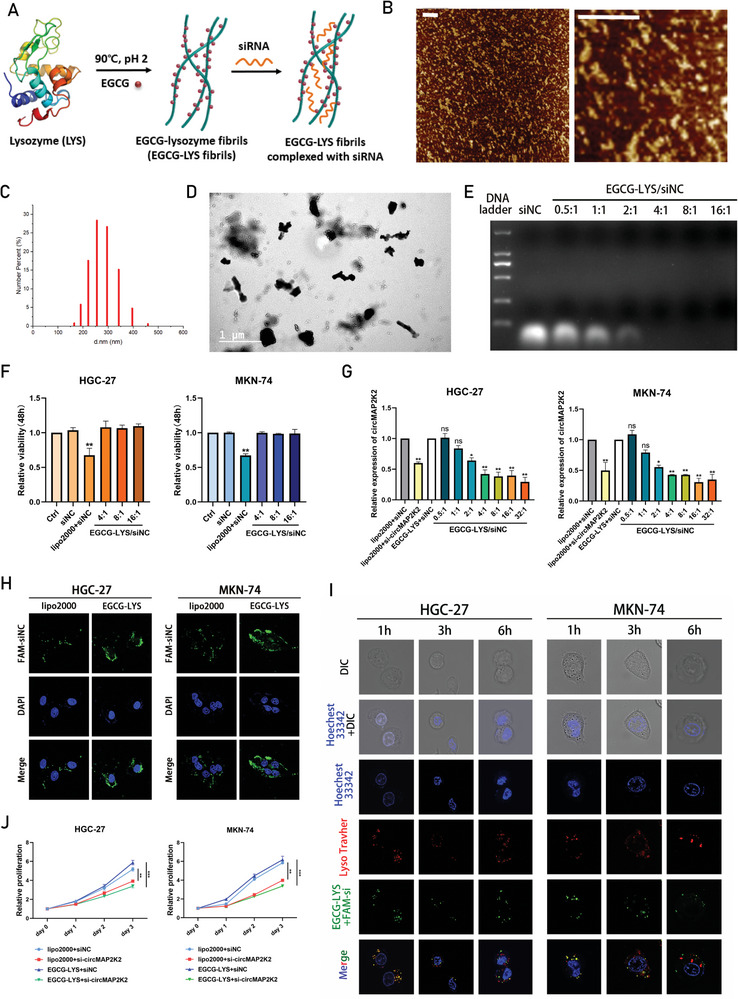
EGCG‐LYS fibrils‐mediated circMAP2K2 silencing synergistically inhibit gastric cancer cell growth in vitro. A) The scheme of EGCG‐LYS fibrils nanocarrier synthesis and siRNA loading. B) AFM particle morphology of EGCG‐LYS fibrils. The left and right images are low/high magnification, respectively (white scale bar, 200 nm). C) Length distribution of carrier particles. The x‐axis is length (nm), and the y‐axis is percentage of particles with certain length. D) TEM particle morphology of EGCG‐LYS fibrils. Horizontally positioned fibrils appear to be curvy long lines, while vertically positioned ones appear to be a dot. E) The binding ability of EGCG‐LYS fibrils to siRNA with different mass ratios; Gel electrophoresis was used for assessment. F) lipo2000 and EGCG‐LYS fibrils were transfected into cells for 48 h, and cytotoxicity was detected by CCK8. Cell viability was calculated as the percentage of living cells relative to untreated control cells. G) The optimal ratio of EGCG‐LYS to siRNA was determined by qRT‐PCR. The expression of circMAP2K2 was used as an indication of transfection efficiency. Lipo2000+NC group was used as control. H) The distribution of lipo2000/FAM‐siNC or EGCG‐LYS/FAM‐siNC in HGC‐27 and MKN‐74 cells was analyzed by confocal microscopy. Green: fluorescein labeled siNC; Blue: DAPI staining of the nucleus. I) Representative confocal images of HGC‐27 and MKN‐74 cells incubated with EGCG‐LYS/FAM‐siNC for 1, 3, and 6 h at 37 °C. Hoechst 33 342 (blue) was used to label the nucleus, Lyso Tracker Red (red) for lysosome, and FAM (green) for siNC. J) Proliferation capacity of HGC‐27 and MKN‐74 cells transfected with lipo2000+siNC, lipo2000+si‐circMAP2K2, EGCG‐LYS+siNC, and EGCG‐LYS+si‐circMAP2K2, respectively. Proliferation rate was normalized to day 0.

Our findings indicated that targeting circMAP2K2 expression may be a promising strategy to reduce tumor proliferation and metastasis. The scheme for preparing EGCG‐LYS fibrils and their complexing with siRNA is presented in **Figure** **7**A. EGCG‐LYS fibrils would form through a one‐step heating process under certain pH conditions, and negatively charged siRNA would be absorbed into the positively charged amnio groups of the EGCG‐LYS fibrils via a simple mixing procedure. The EGCG loading content in EGCG‐LYS fibrils was 0.32 mg mL^−1^. Atomic force microscopy (AFM) images of EGCG‐LYS fibrils showed the coexistence of short fibrils with a relatively small contour length and long fibrils with a relatively large contour length (Figure 7B). The length of EGCG‐LYS fibrils ranged from 15–500 nm and the average contour length was 245±32 nm (Figure 7C). Transmission electron microscopy (TEM) images of EGCG‐LYS fibrils showed rod‐like nanostructures with a high length‐to‐diameter ratio that would facilitate their penetration into cancer cells (Figure 7D). The binding efficiency of EGCG‐LYS fibrils to siRNA was determined by electrophoretic mobility shift assay in agarose gel. The mobility of siRNA in gel decreased with the increase of EGCG‐LYS fibrils in the range of 0.5:1 to 16:1 of the ratio of EGCG‐LYS fibrils to siRNA. The optimal use ratio of EGCG‐LYS fibrils in combination with siRNA was determined to be 4:1 (Figure 7E).

The cytotoxicity of EGCG‐LYS fibrils was examined, and the CCK‐8 assay revealed that treatment of HGC‐27 and MKN‐74 cells with negative control siRNA (siNC) and different doses of EGCG‐LYS/siNC complexes for 48 h did not cause significant changes in cell viability (Figure 7F). The transfection efficiency of EGCG‐LYS fibrils was explored using qRT‐PCR, which showed that the knockdown efficiency of circMAP2K2 was optimal when the ratio of EGCG‐LYS to siRNA was 4:1 (Figure 7G). Fluorescein (FAM)‐labeled siRNA (FAM‐siRNA) and confocal microscopy was used to examine the adsorption of nanoparticle complexes (lipo2000/FAM‐siRNA or EGCG‐LYS/FAM‐siRNA) by HGC‐27 and MKN‐74 cells. Compared with lipo2000/FAM‐siRNA, the EGCG‐LYS/FAM‐siRNA complex showed increased internalization efficiency (Figure 7H). Next, escape efficiency in lysosomes was examined for the EGCG‐LYS‐delivered siRNAs. After incubation for 1 h, the red (LysoTracker) and green (EGCG‐LYS/FAM‐siNC) fluorescence in the cells overlapped, indicating that FAM‐labeled siRNA was encapsulated in the lysosomes. The red and green fluorescence then gradually separated over time, and successful escape of EGCG‐LYS/FAM‐siRNA from lysosomes was observed at 6 h (Figure 7I). These results indicated that EGCG‐LYS/siRNA could achieve gene silencing, and whether EGCG‐LYS/siRNA had the same effect on the biological behavior of cancer cells deserved further investigation. Therefore, the cells were divided into lipo2000+siNC, lipo2000+si‐circMAP2K2, EGCG‐LYS+siNC, and EGCG‐LYS+si‐circMAP2K2 groups for functional experiments. CCK‐8 and wound healing assays revealed that cells co‐transfected with EGCG‐LYS and si‐circMAP2K2 exhibited weaker proliferation and migration ability compared with the other three co‐transfected groups of cells (Figure 7J; Figure S7, Supporting Information).

### 2.9 CircMAP2K2 Silencing Mediated by EGCG‐LYS Fibrils Reduced the Proliferation and Metastasis Ability of Gastric Cancer Cells In Vivo

An equal number of MKN‐74 cells (5 × 10^6^) were injected subcutaneously through the left axillary of nude mice. Until the volume of subcutaneously transplanted tumor was 200 mm^3^. EGCG‐LYS/siRNA complex was injected through the caudal vein every 3 days. After three injections in a row, vital organs and blood samples were collected from each animal immediately after euthanasia. Blood biochemical analysis of liver and kidney function showed that the systemic toxicity of EGCG‐LYS was negligible (Figure S8A, Supporting Information). Moreover, HE staining of the heart, lung, kidney, brain, spleen and liver samples did not reveal significant histological damage, further suggesting there were limited side effects with EGCG‐LYS (Figure S8B, Supporting Information).

**Figure 8:**
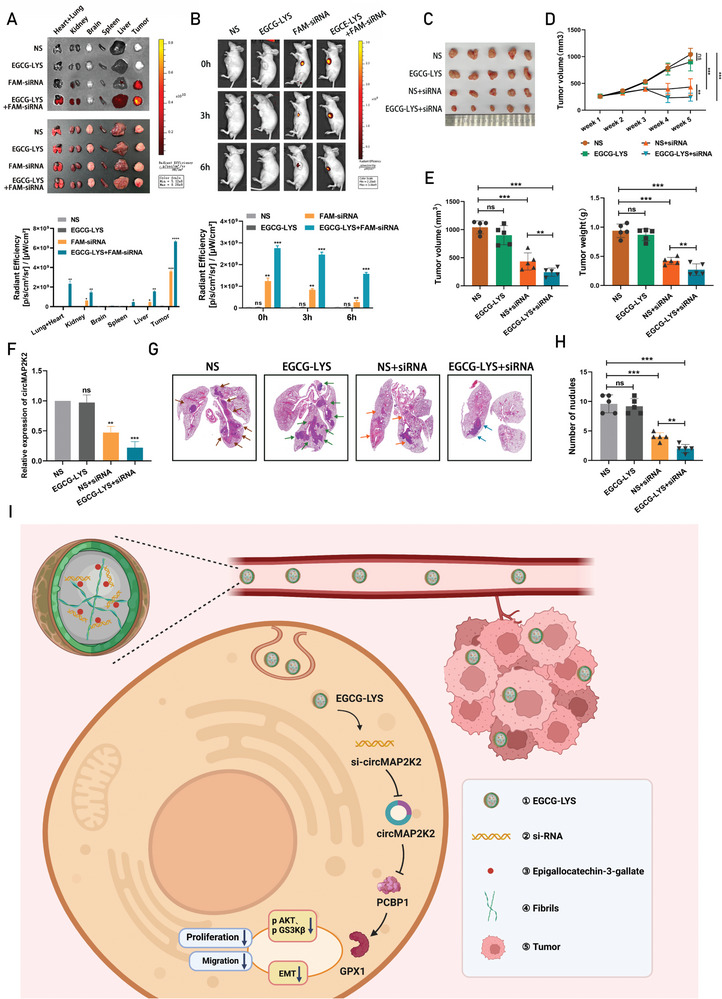
CircMAP2K2 silencing mediated by EGCG‐LYS fibrils reduced the proliferation and metastasis ability of gastric cancer cells in vivo. A) (upper) In vivo imaging of tumor‐bearing nude mice after tail vein injection of NS, EGCG‐LYS, FAM‐siRNA, and EGCG‐LYS+FAM‐siRNA. Fluorescent signals were detected in subcutaneous tumors and different organs, showing the distribution of siRNA. (lower) Quantification of the Fluorescent signals in different organs. B) (upper) In vivo imaging of xenograft mice after intra‐tumoral injection of NS, EGCG‐LYS, FAM‐siRNA, and EGCG‐LYS+FAM‐siRNA. Fluorescent signals were detected at different time points, showing the degradation rate of siRNA. (lower) Quantification of the Fluorescent signals at different time points. C) Representative gross tumor image of subcutaneous xenograft model. D) Weekly changes in tumor volume in each group were recorded and shown. E) Quantification of final tumor volume (left) and mass (right) in the subcutaneous xenograft tumor model. F) Tumor circMAP2K2 expression of different treatment groups in the subcutaneous tumor model were detected by qRT‐PCR. G) Representative images of tumor‐infiltrating lungs of the tail‐vein metastasis model on microscopic HE staining. Arrows indicate metastatic nodules. H) Statistics of the number of metastatic nodules in different groups of the tail‐vein metastasis model. I) Summary of the mechanism by which EGCG‐LYS fibrils‐mediated circMAP2K2 silencing reduced the proliferation and metastasis of gastric cancer cells.

Next, experimentation on mice were devoted to explore the residence time of EGCG‐LYS fibrils carrying siRNA in tumors and whether the nanoparticles are stable in the circulation. Mice bearing gastric cancer tumors were grouped and injected with NS, FAM‐siRNA, EGCG‐LYS, or EGCG‐LYS+ FAM‐siRNA intratumor or via the tail vein, respectively. Compared with the other three groups, the EGCG‐LYS+ FAM‐siRNA complex showed greater accumulation in the heart, lung, kidney, brain, spleen, and liver, especially in tumors after rapidly dissecting out the organs after 6 h after intravenous injection through the tail vein (**Figure** **8**A). This suggests that the nanoparticles would reduce the rate of siRNA clearance from the bloodstream, allowing the siRNA to remain stable in the body for a longer time. The most intense fluorescence was observed in the subcutaneous tumor tissue of mice at 1 h, 3 h, and 6 h after intra‐tumoral injection of EGCG‐LYS+FAM‐siRNA (Figure 8B). Furthermore, the EGCG‐LYS+FAM‐siRNA complex not only has good stability in the circulation in vivo, but also can accurately locate to the tumor site, as proven by the above data. Next, the effect of therapeutic siRNA was tested by dividing MKN‐74 xenograft mice into the following four groups: NS, EGCG‐LYS, NS+si‐circMAP2K2 and EGCG‐LYS+si‐circMAP2K2. When the tumor volume was calculated to be ≈200 mm^3^, different complex was injected into the tumors of mice according to grouping, and tumor size changes were recorded weekly. After 5 weeks, tumors were collected from each animal immediately after euthanasia. CircMAP2K2 silencing mediated by EGCG‐LYS fibrils effectively inhibited tumor growth. The EGCG‐LYS fibrils‐mediated circMAP2K2 knockout mice had a remarkable reduction in the overall mean tumor volume (Figure 8C). In comparison with the NS+si‐circMAP2K2 group, the tumor volume in the EGCG‐LYS+si‐circMAP2K2 group showed more significant growth inhibition at the later stage of tumor growth (Figure 8D). This may be due to the greater tumor‐targeting ability of nanodrugs with smaller molecules. The data also showed that circMAP2K2 silencing mediated by EGCG‐LYS fibrils could effectively inhibit tumor growth, as shown by significantly reduced tumor volume and weight compared with the other three groups (Figure 8E). Tumor sections were subjected to qRT‐PCR and immunohistochemical staining to analyze the expression of circMAP2K2 (Figure 8F), PCBP1 and GPX1 (Figure S9, Supporting Information) in the xenograft tumors.

In addition to actively treating the primary tumor, fighting cancer metastasis is also a focus of anticancer therapy. Inspired by this, mice were intravenously injected with wild‐type MKN‐74 cells to establish tumor metastasis models. Four weeks later, NS, EGCG‐LYS, si‐circMAP2K2, and EGCG‐LYS+si‐circMAP2K2 were injected into the tail vein of mice. Consistent with the hypothesis, there were significantly fewer lung metastasis sites in the EGCG‐LYS+si‐circMAP2K2 group compared with the other groups, which was reflected in the HE staining of lung sections from the mice (Figure 8G,H). In summary, these anticancer in vivo experiments reveal the clinical potential of EGCG‐LYS fibrils‐mediated si‐circMAP2K2 nanomedicine.

## 3 Discussion

In this study, a novel circRNA (circMAP2K2) with high expression in gastric cancer was initially discovered by screening two circRNA microarray data sets in the GEO database. The existence of circMAP2K2 in gastric cancer cells was proven by Sanger sequencing, RNase R treatment, actinomycin D treatment, and FISH. Moreover, circMAP2K2 was confirmed to promote the malignant biological behaviors of proliferation and metastasis of gastric cancer cells through gene silencing or overexpression experiments. To our knowledge, circMAP2K2 has never been studied in gastric cancer, indicating that this research provides novel insights of the mechanism of circRNA in gastric cancer.

CircRNAs can exert several functions in normal and cancer cells. Some of the most well‐studied mechanism included RNA‐binding proteins (RBPSs) interaction, miRNA sponge and novel protein coding.^[^ ^33^ ^]^ Among these important functions, the interaction between circRNA and RNA‐binding protein is quite unique and indispensable. Because not only can circRNA interact with RBPs to regulate the expression of downstream genes, but also some RBPs can directly bind to pre‐RNA to assist the back‐splicing and synthesis of circRNAs.^[^ ^34^ ^]^ To further investigate the potential RBPs that may interact with circMAP2K2, mass spectrometry detection results after RNA pull‐down assays and the intersection prediction of the RBPmap database were utilized to obtain the potential binding proteins that may interact with circMAP2K2. A series experiments confirmed that PCBP1 could interact with circMAP2K2. PCBP1 is an RBP that is ubiquitously expressed in human cells and is considered as a potential tumor suppressor for many types of cancers, including gastric cancer.^[^ ^31^, ^35^, ^36^, ^37^ ^]^ Mechanism study discovered that circMAP2K2 can disrupt the protein stability of PCBP1 through proteasomes degradation, and the alteration of PCBP1 further affect the expression of GPX1. GPX1 is considered as the main enzyme in the defense against reactive oxygen species as it regulates the normal supply of glutathione, which is constantly supplied by the activity of glutathione reductase, and has a detoxification effect on hydrogen and lipid peroxides to reduce oxidative damage to DNA, proteins, and lipids.^[^ ^38^ ^]^ Furthermore, there have been several reports that GPX1 inhibits the development of tumors in gastric and pancreatic cancer,^[^ ^32^, ^39^ ^]^ and one study highlights that GPX1 is responsible for EMT inhibition by regulating the AKT/GSK3β/Snail signaling axis.^[^ ^39^ ^]^ This was further confirmed in our results, whereby GPX1 silencing promoted an EMT‐like phenotype in gastric cancer cells via the AKT/GSK3β signaling pathway. On account of the above basic experimental results, we conclude that circMAP2K2 is a potential key biological target for the treatment of gastric cancer. However, there are still some problems yet to be addressed in this study. For example, how circMAP2K2 affects the degradation of PCBP1 protein is still unknown. Is it related to the E3 ubiquitin ligase? Also, how PCBP1 regulates the expression of GPX1 is yet to be discovered. Further research is required in order to solve these problems.

As mentioned above, RBPs can both mediate the downstream pathway of circRNA and the biogenesis of circRNA itself. Our study also revealed that the EIF4A3, a classic RBP with an essential role in RNA splicing,^[^ ^40^, ^41^ ^]^ can bind to the upstream region of circMAP2K2 mRNA transcripts and induce its expression. Many previous circRNAs studies provide knowledge on how differentially‐expressed circRNAs regulate cancer progression via different functional pathways, but only few of these studies explain why the circRNAs express differentially at the first place. Our research discovered that EIF4A3 participate in the formation of circMAP2K2. However, the exact mechanism of how circMAP2K2 is induced is yet to be elucidated.

Exogenous expression of cancer‐suppressive ncRNAs or knockout of carcinogenic ncRNAs by siRNAs have been studied to reverse gastric cancer chemo‐resistance.^[^ ^29^ ^]^ The combination of traditional chemotherapy or targeted therapy with interventional therapy of ncRNAs may be a new approach to solve the problem of drug resistance in patients with advanced gastric cancer. As a cell‐based mechanism for inhibiting gene expression, RNAi offers new ideas for drug development.^[^ ^15^, ^19^ ^]^ Consequently, there have been increasing efforts to identify novel disease biomarkers and related ligands for targeted drug delivery applications. To date, more than 24 nanotechnology therapeutic products have been approved for clinical use.^[^ ^42^ ^]^ New treatments enabled by nanotechnology are not yet widely used in clinical practice, but we believe that nanotechnology will have advantages in many important areas in the future.

As a nanocarrier, EGCG‐LYS has many superior characteristics that makes it a successful therapeutic method. EGCG‐LYS contains positively charged amniotic membrane group, which is critical for its siRNA absorption and tumor targeting function. Because siRNA is negative charge, simple incubation can readily generate EGCG‐LYS‐siRNA complex through electrostatic interactions. Also, many malignant tumors cells display an overall negative surface charge, mainly due to the hypermetabolic status of cancer cells and expelling anionic waste, while normal cells are usually neutral on their surface.^[^ ^25^ ^]^ This discrimination allows EGCG‐LYS to preferentially attach tumor cells, i.e., specific tumor targeting, thus less undesirable off‐target effect. Moreover, when EGCG‐LYS‐siRNA enters tumor cell, other negatively charged components in the cytoplasm would compete with siRNA for EGCG‐LYS fibrils binding, resulting in release of siRNA from EGCG‐LYS fibrils. Beside siRNA absorption and tumor targeting, LYS fibrils also have strong mechanical strength, which allows them to maintain their stable structure under harsh conditions such as low pH and long‐term heat treatment.^[^ ^23^ ^]^ This makes LYS fibrils achieve high circulation stability and siRNA delivery efficiency. In our experiment conducted in vivo, circMAP2K2 silencing mediated by EGCG‐LYS fibrils could effectively inhibit tumor growth and metastasis, especially the tumor proliferation inhibition effect at the advanced stage of treatment. Finally, both LYS and EGCG are natural compounds,^[^ ^21^ ^]^ meaning that at normal dosage, these nanocarriers at least will not cause significant toxicity to the whole body. Our animal experiment also confirmed that the nanoparticles had good biological safety in vivo, no damage to vital organs was found in the short‐term. The purpose of EGCG‐LYS fibrils to deliver si‐circMAP2K2 to target organs and tissues represents a breakthrough in this field and shows great promise as a treatment option for gastric cancer diseases.

## 4 Conclusion

In conclusion, according to our above findings, the overexpression of circMAP2K2 induced by EIF4A3 in GC is related to the mechanism of promoting cancer. CircMAP2K2 regulates the PCBP1/GPX1 axis through proteasome‐mediated degradation, resulting in an EMT‐like phenotype, and activated AKT/GSK3β signaling pathway enhances proliferation and metastasis of GC. In addition, EGCG‐LYS/si‐circMAP2K2 nanocarrier delivery system of small interfering RNA can effectively and safely deliver siRNA system to the tumor and has the function of knocking down target genes, providing a new tool and approach for the treatment of gastric cancer.

## 5 Experimental Section

### Cell Lines and Culture Conditions

The human gastric cancer cell lines HGC‐27, MKN‐74, and MKN‐45 were purchased from the Chinese Academy of Sciences. All three cell lines were cultured in RPMI 1640 medium (GIBCO BRL, Grand Island, NY, USA) supplemented with 10% fetal bovine serum (FBS, PAN‐Seratech, Germany) and 1% penicillin/streptomycin (Invitrogen, Carlsbad, CA, USA) at 5% CO_2_ and 37 °C.

### Bioinformatics Analysis

Two microarray data sets were retrieved from the GEO repository by searching the keywords “circRNA” and “gastric cancer”. Dataset GSE93541 comprises circRNA expression profiles for six human plasma samples derived from patients with gastric cancer; dataset GSE83521 comprises transcriptional profiling of circular RNA in stage III gastric cancer patients: six tumors versus six normal mucosa tissues. R (version 3.4.3) (https://www.r‐project. org/) was used for subsequent data analysis.

### Plasmid Constructs, siRNA Interference, and shRNA Transfection

CircMAP2K2 targeting siRNA and control siRNA were synthesized by RiboBio (Guangzhou, China). Short hairpin RNA (shRNA) and overexpression plasmids targeting circMAP2K2, PCBP1, and GPX1 were purchased from Gene Chemical Corporation (Shanghai, China). SiRNA sequences were listed Table S1 (Supporting Information).

Cells were seeded in 6‐well plates and cultured overnight. For gene silencing, siRNA was transfected into the cells with lipo2000 (Invitrogen) or EGCG‐LYS after replacing the medium with fresh medium. shRNA lentivirus was transfected with Polybrene (Merck Millipore, Germany). After incubation for 8 h, the culture medium was discarded, the cells were washed with PBS, and fresh medium was added for a further 48 h. In the overexpression experiment, cells were seeded in 6‐well plates and cultured overnight, then lipo3000 (Invitrogen) was used to transfect overexpression plasmids into the cells and polybrene was used to transfect overexpression lentivirus into the cells. After incubation for 8 h, the culture medium was discarded, the cells were washed with PBS, and fresh medium was added for a further 48 h. The success of gene silencing or overexpression was verified by qRT‐PCR.

### RNA and Genomic DNA Extraction

Cells were treated with TRIzol (Invitrogen) and total RNA was extracted. A genomic DNA isolation kit (Sangon Biotech, Shanghai, China) was used to extract genomic DNA (gDNA) from cells.

### RNA Processing and Quantitative Real‐Time PCR

Total RNA (2 µg) was divided into two groups: with or without 3 U µg^−1^ RNase R (Epicentral Technologies, USA), and incubated at 37 °C for 30 min, and purified product RNA was obtained using the RNeasy kit (Qiagen, Germany) after the 30 min incubation. Nucleic acid quantification was performed using a nanophotometer (IMPLEN, Germany), followed by reverse transcription to synthesize cDNA. Gene expression was measured by quantitative real‐time PCR (qRT‐PCR) using the SYBR Green qPCR Mix (EZBioscience, USA) according to the manufacturer's instructions. qRT‐PCR was performed on a QuantStudio5 RT‐PCR system (ThermoFisher Scientific). Primers used for PCR and qRT‐PCR were shown in Table S1 (Supporting Information). GAPDH was used as a reference for mRNA/circRNA expression.

### Determination of Actinomycin D

Where appropriate, 2 µg mL^−1^ actinomycin D (Sigma, USA) was added into the cell culture medium and the cultures were terminated at different time points. Cell precipitation was collected, RNA was extracted, and the stability of the RNA was detected by qRT‐PCR, as described above.

### Determination of Actinomycin D–Fluorescence In Situ Hybridization (FISH)

The modified oligonucleotide probe sequence of circMAP2K2 was synthesized by RiboBio. A fluorescent in situ hybridization kit (RiboBio) was used to hybridize the probe with cells, following the manufacture's instruction. Images of the cells were captured and analyzed using a confocal laser scanning microscope (FV1000, Olympus, Japan).

### Agarose Gel Electrophoresis

Tris‐acetate (TAE) buffer (1×, Biosharp, Beijing, China) was used to prepare agarose gels with a concentration of 1.2%. Nucleic acids (DNA or RNA) were electrophoresed at 140 V for 30 mins and a UV gel imaging system (UVP GelStudio PLUS touch, Germany) was used to observe and photograph gel bands.

### Analysis of Cell Proliferation

Cell viability was detected with a CCK‐8 kit (#HY‐K0301, MCE, China). Briefly, 2 × 10^3^ cells per well were seeded in a 96‐well plate, with three replicates per group. Cell viability was tested on day 0, 1, 2, and 3 by adding 10 µL CCK‐8 solution to each well, incubating the plates for 2 h, 37 °C, and then measuring the absorbance at 450 nm.

For EdU experiment, cells were seeded in 6‐well plates (with cover glass) at 2 × 10^5^ cells well^−1^ and incubated with different treatments for 48 h. The cultured cells were permeated with EdU (5‐ethynyl‐2′‐deoxyuridine) using the BeyoClick EdU‐594 Cell Proliferation Detection Kit (Beyotime, China) according to the manufacturer's instructions. Briefly, the final concentration of EdU was adjusted to 10 µM (working solution). The working solution was added to gastric cancer cells and continued to culture for 1 h, followed by fixation with 4% paraformaldehyde (Beyotime) for 15 min and penetration with 0.3% Triton X‐100 PBS. The cells were washed with PBS and reaction solution was added for 30 min incubation. The nuclei were stain with DAPI dye. The wave length for EdU detection was 594 nm. The final results were imaged with an automatic inverted fluorescence microscope (IX83, Olympus, Japan).

### Wound‐Healing Assay

Cells were inoculated in 6‐well plates and incubated for 48–72 h after transfection. A 1000 µL sterile pipette tip was then used to create a scratch of constant width in the center of each well. The cells were incubated in serum‐free medium to exclude their proliferation effect. Images were taken with an inverted microscope (Olympus) at 0 and 48 h to measure cell migration and movement.

### Cell Migration and Cell Invasion Experiments

Cell starvation treatment was performed prior to detection by incubating cells in serum‐free medium for 8 h. The cells were re‐suspended in serum‐free medium, adjusted to 1 × 10^5^ cells in 100 µL, and then the cell suspension was added to a 24‐well transwell plate (Corning, USA). For invasion assays, 2% Matrigel (Corning, USA) was used to coat the membrane. Medium containing 10% FBS was used as the chemoattractant in the lower compartment. Cells were incubated for 48 h in migration and invasion experiments. Finally, the upper chamber was fixed with 4% paraformaldehyde (Beyotime), air‐dried at room temperature, and then stained with 0.4% crystal violet (Beyotime) for 20 min. The cells on the upper surface were wiped with a cotton swab, observed through a randomly selected mirror field under an Olympus IX83 inverted microscope, and the number of invaded/migrated cells was calculated.

### RNA Pull‐Down Assay

A Pierce Magnetic RNA‐Protein Pull‐Down Kit (Thermo Fisher Scientific, MA, USA) was used to perform RNA pull‐down experiments. A biotinylation probe was designed for the circMAP2K2 junction region (RiboBio). Oligo probe (RiboBio) was used as negative control. Both probes were incubated at room temperature for 2 h with a mixture of streptavidin magnetic beads (Invitrogen). After washing to remove unbound/non‐specific binding beads, the cell lysate was mixed with RNA beads and incubated overnight at 4 °C. RNA‐binding proteins (RBPs) were then isolated by sodium dodecyl‐sulfate polyacrylamide gel electrophoresis (SDS‐PAGE) and stained with silver to observe the differential bands. The differential proteins were subsequently analyzed by mass spectrometry.

### RNA Pull‐Down Assay–RNA Immunoprecipitation (RIP)

RIP was detected using a Magna RIP RNA‐Binding Protein Immunoprecipitation Kit (Millipore, MA, USA). Briefly, 5 µg specific PCBP1 and PCBP2 antibody was incubated with protein A/G beads at room temperature for 30 min. Next, 100 µL cell lysis solution was added and incubated overnight at 4 °C. The coprecipitated RNA was then extracted by TRIzol, and the content of circMAP2K2 was detected by qRT‐PCR.

### In Vitro Ubiquitination Assay

HGC‐27 and MKN‐74 cells were transfected with His‐labeled ubiquitin (His‐UB) plasmid. After transfection for 48 h, 20 µM MG132 was added to the cells for 6 h. Next, the cells were resuspended with buffer A (6 M guanidine HCL, 0.1 M disodium hydrogen phosphate, 0.1 M sodium dihydrogen phosphate, 10 mM imidazole, pH 8.0) and cell lysate precipitates were obtained by ultrasound and centrifugation, to which 50 µl Ni‐NTA magnetic beads were added, mixed, and incubated at room temperature for 3 h. The pull‐down product attached to the beads was washed twice with buffer A, once with buffer A mixed with buffer TI (25 mM Tris‐HCl, 20 mM imidazole, pH 6.8) in a ratio of 1:3, and then twice with buffer TI. Western blotting was used to analyze the His‐UB conjugate protein pulled down by Ni‐NTA magnetic beads.

### Preparation of EGCG‐LYS Fibrils

Lysozyme (1.0 g) was dissolved in deionized water (50 mL) to obtain lysozyme solution. Then, EGCG (20 mg), dissolved in 2 mL water, was added into the solution. The mixture was stirred at 300 rpm at 90 °C for 12 h after adjusting to pH 2 with HCl. Subsequently, the mixture was cooled in ice‐water and subjected to dialysis for 3 days (dialysis membrane molecular weight cutoff: 14 kDa). The obtained EGCG‐LYS fibrils suspension was stored at 4 °C for further use.

### Characterization of EGCG‐LYS Fibrils

EGCG (purity≥95%) was obtained from BSZH Science (Beijing, China). Hen egg white lysozyme (20000 U mg^−1^) was obtained from Beijing Solarbio Science & Technology (Beijing, China). The EGCG‐LYS fibrils suspension was diluted 1:50 in deionized water before characterization. The morphologic properties of EGCG‐LYS fibrils were analyzed by AFM and TEM. Sample suspension was dripped onto freshly cleaved mica surface and dried by nitrogen gas. AFM images were obtained by a Multimode Nanoscope‐V (Veeco instruments, USA) using tapping mode. A silicon tip with a nominal spring constant of 5 N m^−1^ was used and the contour length distribution of EGCG‐LYS fibrils was acquired after analyzing at least 100 individual fibrils from AFM images. Samples for TEM analysis were prepared by spreading EGCG‐LYS fibrils suspension onto a carbon‐coated microscope grid and drying at room temperature. The grid was then observed using a Tecnal‐10 transmission electron microscope (Philips, Netherlands) under accelerating voltage of 200 kV. EGCG content in EGCG‐LYS fibrils was analyzed by measuring the amount of free EGCG in the dialysis medium using a high‐performance liquid chromatography (HPLC) system and regarding the free EGCG as unloaded EGCG to obtain the amount of EGCG in the EGCG‐LYS fibrils.

### Preparation and Binding‐Efficiency of EGCG‐LYS Fibrils/siRNA

For siRNA loading, the volume ratio of EGCG‐LYS fibrils suspension (prepared as described above) to siRNA (1 µl, 10 nmol) ranged from 0.5:1 to 16:1. Then mixed well and incubated at room temperature for 1 h. Then, all samples were adjusted to 20 µl. The siRNA loading efficiency was subsequently evaluated by gel electrophoresis.

### Preparation and Binding‐Efficiency of EGCG‐LYS Fibrils/siRNA–Immunohistochemical (IHC)

To perform IHC staining, the sections were first immersed in citrate solution (Solarbio) and heated to boil for antigen retrieval. Then, the sections were treated with 3% hydrogen peroxide (Solarbio) to inactivate endogenous peroxidase. 5% bovine serum albumin (Beyotime) was added to the section and incubated for 1 h. Primary antibodies were then added to the sections and incubated overnight at 4 °C. After washing with PBS, secondary antibodies were added, and continued to incubate for 1 h at room temperature. Finally, the signal was detected with a 3,3′‐diaminobenzidine (DAB) substrate kit (Zhongshan Jinqiao, China) and the nuclei were stained with hematoxylin (Solarbio).

### Animal Experiment

All animal care and experimental procedures were performed in accordance with National Institutes of Health guidelines and approved by the Institutional Animal Care and Use Committee of Sun Yat‐sen University (SYSU‐IACUC‐2022‐001042). BALB/c nude mice aged 4–5 weeks were purchased from GemPharmatech (Nanjing, China). The MKN‐74 cell line was used to establish cell lines stably transfected with control vector and circMAP2K2 shRNA, with eight mice in each group. Mice were subcutaneously injected with an equal volume of cells (5×10^6^) transfected with control vector or shRNA through the left axilla. Tumor size was measured once a week. Four weeks later, all mice were euthanized, subcutaneous tumors were dissected and weighed, and subsequent detection experiments were performed.

For lung metastasis experiments, eight mice were set in each group. Cells transfected with control vector or circMAP2K2 shRNA (1 × 10^6^) were injected via the tail vein. All mice were euthanized after 8 weeks, and lung tissues were collected and stained with HE. An Olympus IX83 inverted microscope was used to capture images of sections to count lung metastases in each sample. The severity of metastasis was assessed by counting the number of pulmonary Nodules to test the therapeutic siRNA effect, BALB/c male nude mice aged 4–5 weeks were randomly divided into four groups (10 nmol siRNA and 4 µl EGCG‐LYS in 50 µl normal saline was injected into tumor or in 100 µl normal saline was injected via tail vein): NS group, EGCG‐LYS group, NS+si‐circMAP2K2 group, and EGCG‐LYS+ si‐circMAP2K2 group. The mice were intravenously or subcutaneously injected with MKN‐74 cells. After the tumor volume reached ≈200mm^3^/ bearing MKN‐74 cell tumors for 4 weeks, different nanoparticle/siRNA complexes were administered through intra‐tumoral or caudal veins routes at 4‐day intervals, and changes in tumor size were recorded weekly. Five weeks later, tumors, and lungs were collected to prepare for HE staining from each animal immediately after euthanasia. Image acquisition was performed with an automatic inverted fluorescence microscope (Olympus IX83).

### Internal Distribution of Delivery Materials

BALB/c male nude mice aged 4–5 weeks were purchased from GemPharmatech (Nanjing, China) and randomly divided into 4 groups with 3 mice in each group. Mice were injected subcutaneously (5 × 10^6^) with an equal number of MKN‐74 cells through the left axilla. FAM‐siNC was used as FAM‐siRNA in this part. When the tumor volume reached 200 mm^3^, NS, EGCG‐LYS, NS+FAM‐siRNA, and EGCG‐LYS+FAM‐siRNA were then injected through intravenous or intra‐tumoral routes (n = 3 in each group). The mice were euthanized 6 h after injection, tumor, organ, and blood samples were collected, and complex biological distributions were detected using the Xenogen IVIS Lumina system (IVIS spectroscopy, PerkinElmer).

### Statistical Analysis

All tests were performed in triplicate, and the resulting data were presented as (mean ± standard deviation). Statistical analysis of the data was performed using SPSS 25.0 and GraphPad Prism 8.0. Unpaired two‐tailed Student's t test was used for comparison of two groups; one‐way ANOVA and Bonferroni test for three or more groups. *P* <0.05 was considered as being statistically significant. * *p* <0.05, ** *p* <0.01, *** *p* <0.001 indicate different degrees of statistical significance.

## Conflict of Interest

The authors declare no conflict of interest.

## Author Contributions

J.D., Z.Z., M.Z., Y.W., and J.C. contributed equally to this work. Conceptualization: J.D., M.Z., and Y.L. Data curation: J.D., M.Z., and Y.L. Formal analysis: M.Z., Z.Z., Y.W., J.C., and J.C. Funding acquisition: J.C. and T.C. Investigation: T.Y., Y.X., and G.S. Methodology: Z.Z., Y.W., and J.C. Project administration: Y.L. Resources: J.C., X.L., and Y.L. Software: J.C., J.C., and G.S. Supervision: J.C., X.L., and Y.L. Validation: T.Y., Y.X., and G.S. Visualization: J.C. and Y.L. Writing – original draft: J.D. Writing – review & editing: J.C, X.L., and Y.L.

## Supporting information

Supporting Information

Supporting Information

Supporting Information

## Data Availability

The data that support the findings of this study are available from the corresponding author upon reasonable request.
