# Supplementary material for: EGCG‐LYS Fibrils‐Mediated CircMAP2K2 Silencing Decreases the Proliferation and Metastasis Ability of Gastric Cancer Cells in Vitro and in Vivo
Source: Adv Sci (Weinh). 2023 Sep 26;10(32):2304075. doi: 10.1002/advs.202304075 (PMC10646246; doi:10.1002/advs.202304075)
Supplement: Supplementary file 1 — Supporting Information [file ADVS-10-2304075-s003.pdf]

ADVANCED SCIENCE
Open Access

Supporting Information

for *Adv. Sci.*, DOI 10.1002/advs.202304075

EGCG-LYS Fibrils-Mediated CircMAP2K2 Silencing Decreases the Proliferation and Metastasis Ability of Gastric Cancer Cells in Vitro and in Vivo

Jiaqi Dong, Zhousan Zheng, Mi Zhou, Yunfei Wang, Jiajie Chen, Junjie Cen, Tiefeng Cao, Taowei Yang, Yi Xu, Guannan Shu, Xuanxuan Lu and Yanping Liang**

Supplementary information for

EGCG-LYS fibrils-mediated circMAP2K2 silencing decreases the proliferation and metastasis ability of gastric cancer cells *in vitro* and *in vivo*

Authors

Jiaqi Dong, Zhousan Zheng, Mi Zhou, Yunfei Wang, Jiajie Chen, Junjie Cen, Tiefeng Cao, Taowei Yang, Yi Xu, Guannan Shu, Xuanxuan Lu, Yanping Liang

Corresponding authors: Xuanxuan Lu (luxuanxuan2@jnu.edu.cn) and Yanping Liang (liangyp9@mail.sysu.edu.cn).

This docx file contains supplemental **Figure S1 to S9**.

For supplemental tables, please refer to individual .xlsx files.

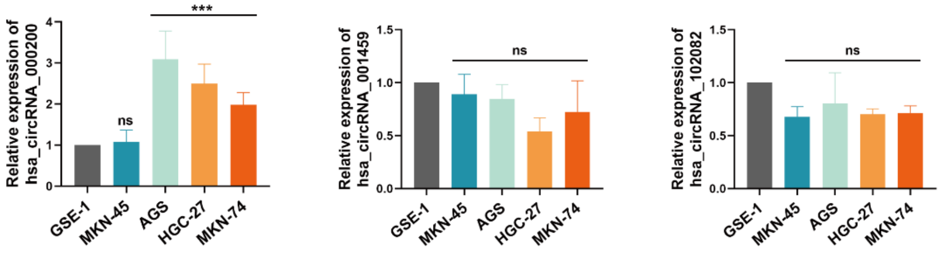

Figure S1. The abundance of hsa_circRNA_000200、hsa_circRNA_001459、hsa_circRNA_102082 in normal gastric epithelial cell lines GSE-1 and gastric cancer cell lines MKN-45, AGS, MKN-74 and HGC-27 was determined by qRT-PCR

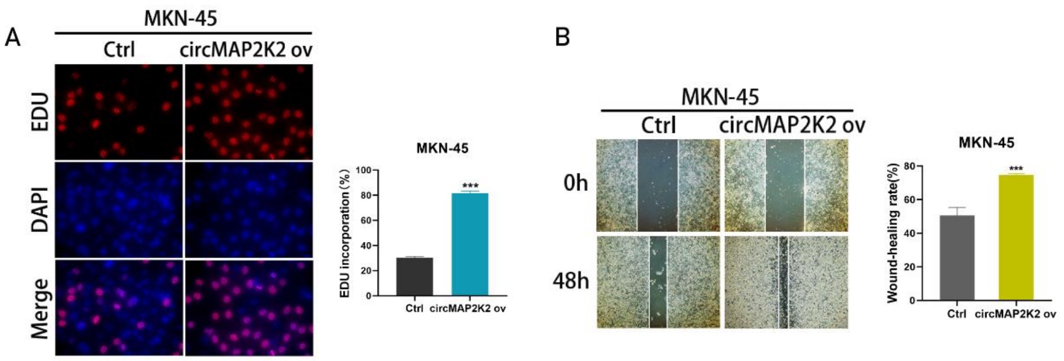

Figure S2. A. EdU assay and **B.** wound healing assay were used to detect MKN-45 cells transfected with circMAP2K2 overexpression plasmid or control (Ctrl) vector.

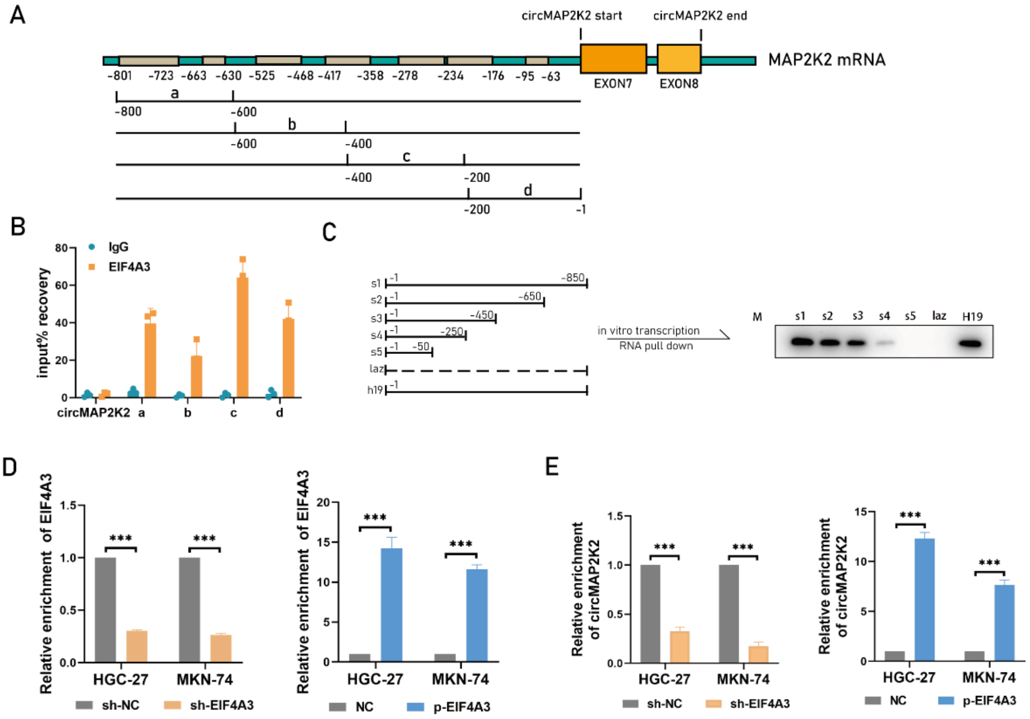

Figure S3. A. The binding sites of EIF4A3 in the upstream region of the MAP2K2 mRNA transcript was predicted using circRNA interactome. **B.** The binding of EIF4A3 to MAP2K2 pre-mRNA was verified by RIP assay. CircMAP2K2 was used as a negative control. qRT-PCR was used to measure the abundance of transcripts relative to the input amount. **C.** Five RNA constructs were truncated to varying degrees (s1-s5) and contained EIF4A3 binding sites. Laz was used as a negative control for nonsense sequences, and H19 was used as a positive control. RNA pull-down assay was used to analyze the interaction between EIF4A3 and circMAP2K2 mRNA. **D-E.** The expression of circMAP2K2 in HGC-27 and MKN-74 cells after EIF4A3 up-regulation or down-regulation was detected by qRT-PCR.

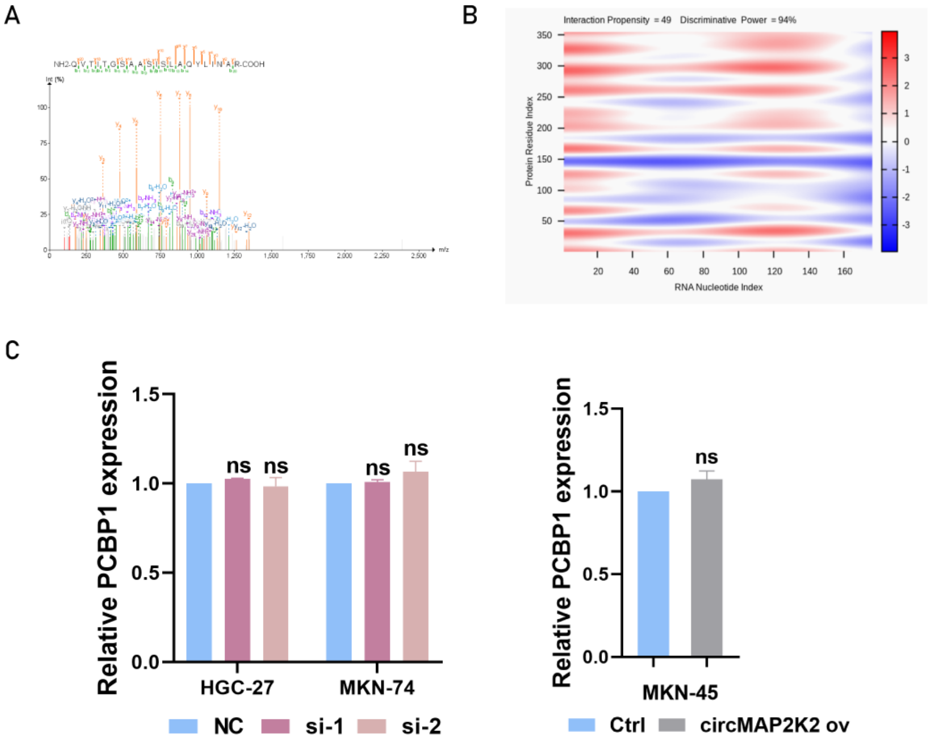

Figure S4. A. Mass spectrometric secondary structure of PCBP1. **B.** Predicted binding sites of circMAP2K2 and PCBP1 proteins (catRAPID figure). **C.** qRT-PCR show the mRNA level and protein level of PCBP1 after circMAP2K2 knockdown and overexpression.

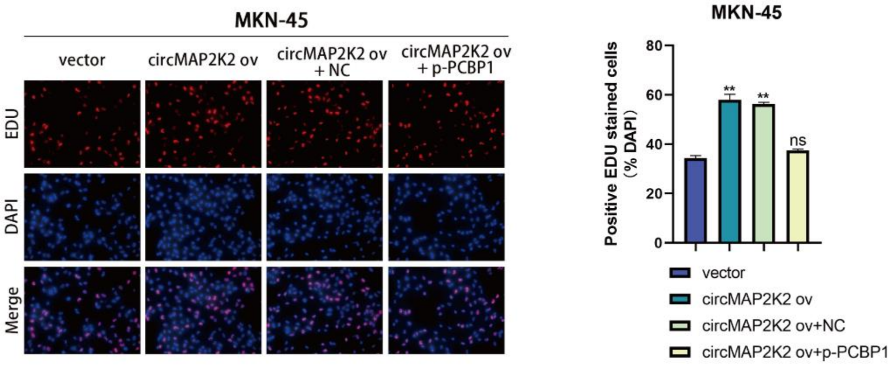

Figure S5. EUD showed that overexpression of PCBP1 eliminated the stimulative effect of circMAP2K2 up-regulation in MKN-45 cells.

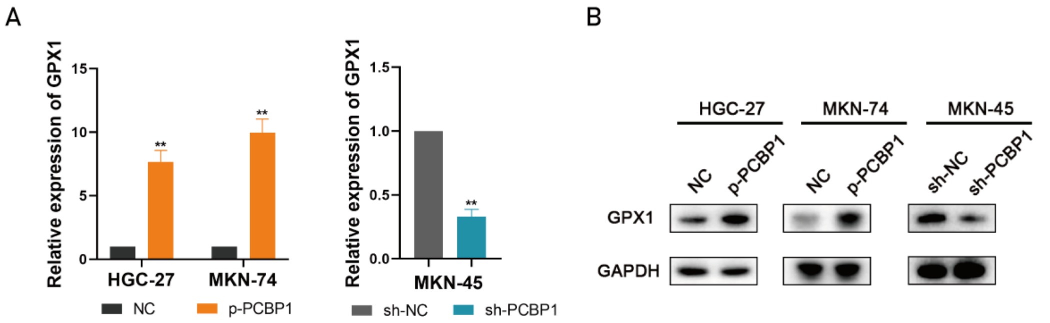

Figure S6. A. qRT-PCR and **B.** Western blot analysis were used to detect the proportional relationship between PCBP1 and GPX1.

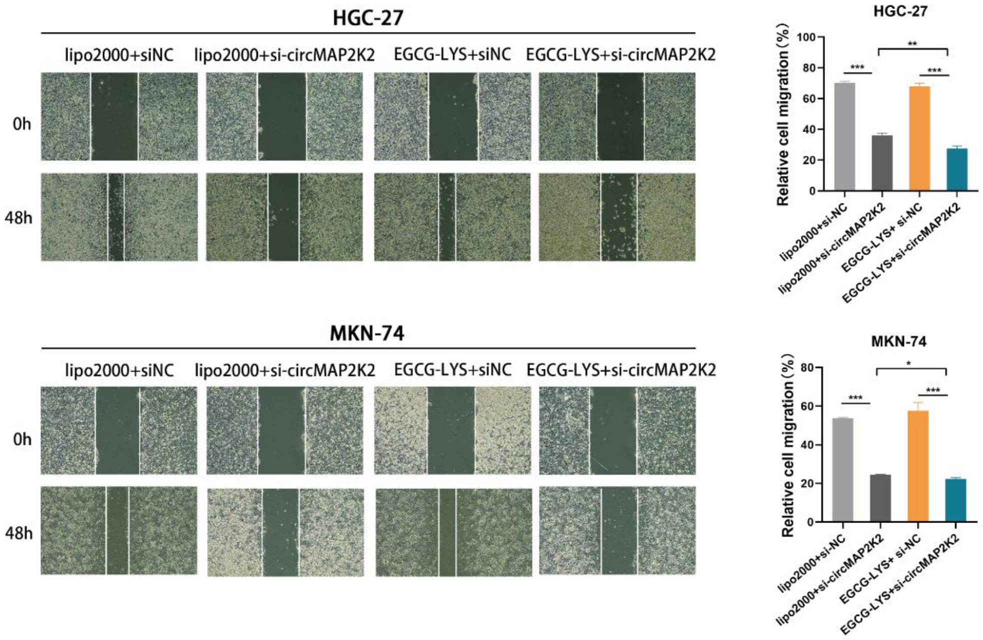

Figure S7. The results of wound healing assay showed that the migration ability of HGC-27 and MKN-74 cells transfected with lipo2000+siNC, lipo2000+si-circMAP2K2, EGCG-LYS+ siNC and EGCG-LYS+si-circMAP2K2, respectively.

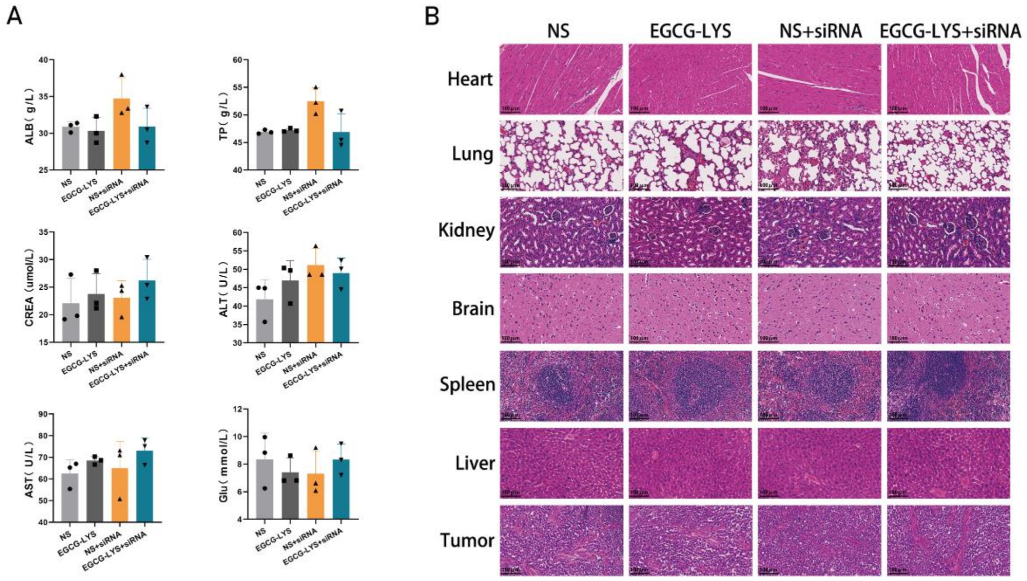

Figure S8. A. After three consecutive injections of NS, EGCG-LYS, free si-circMAP2K2 or EGCG-LYS+si-circMAP2K2, Serum albumin (ABL), total protein (TP), creatinine (CREA), alanine aminotransferase (ALT), glutamine aminotransferase (AST), and glucose (Glu) levels. **B.** After three consecutive injections of NS, EGCG-LYS, free si-circMAP2K2 or EGCG-LYS+ si-circMAP2K2, after that, histological sections of the major organs were obtained.

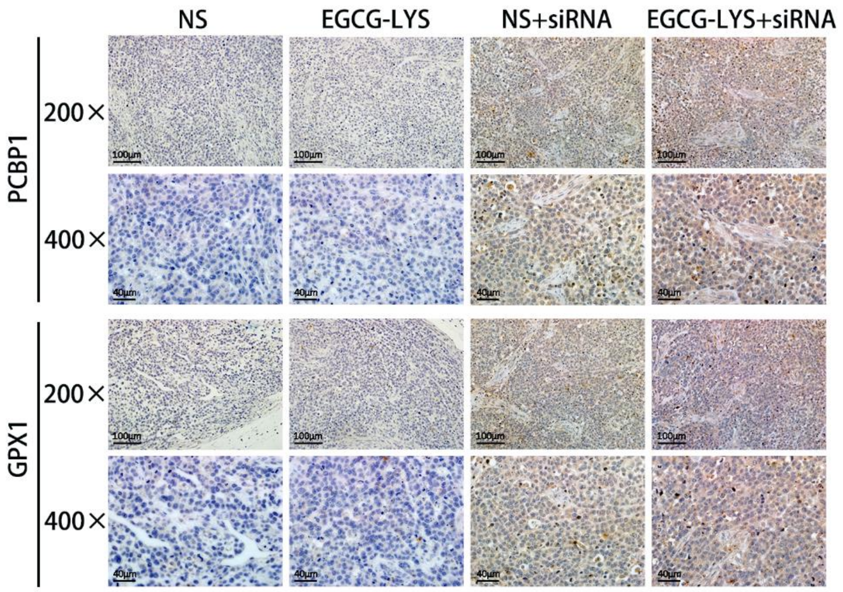

Figure S9. Immunohistochemical staining of tumors derived from subcutaneous xenografts.
